# FK866 attenuates acute hepatic failure through c-jun-N-terminal kinase (JNK)-dependent autophagy

**DOI:** 10.1038/s41598-017-02318-7

**Published:** 2017-05-19

**Authors:** Enshuang Guo, Renlong Li, Jiankun Yang, Jun Zhang, Anyi Li, Yan Yang, Shenpei Liu, Anding Liu, Xiaojing Jiang

**Affiliations:** 1 Graduate School, Southern Medical University, 1023 Shatai Nan Road, Guangzhou, 510515 China; 2 Department of Infectious Diseases, Wuhan General Hospital, 627 Wuluo Road, Wuhan, 430070 China; 3 Experimental Medicine Center, Tongji Hospital, Tongji Medical College, Huazhong University of Science and Technology, 1095 Jiefang Avenue, Wuhan, 430030 China; 4 Animal Experiment Center, Tongji Hospital, Tongji Medical College, Huazhong University of Science and Technology, 1095 Jiefang Avenue, Wuhan, 430030 China

## Abstract

FK866 exhibits a protective effect on D-galactosamine (GaIN)/lipopolysaccharide (LPS) and concanavalin A (ConA)-induced acute liver failure (ALF), but the mechanism by which FK866 affords this benefit has not yet been elucidated. Autophagy has a protective effect on acute liver injury. However, the contribution of autophagy to FK866-conferred hepatoprotection is still unclear. This study aimed to investigate whether FK866 could attenuate GaIN/LPS and ConA-induced ALF through c-jun-N-terminal kinase (JNK)-dependent autophagy. *In vivo*, Mice were pretreated with FK866 at 24, 12, and 0.5 h before treatment with GaIN/LPS and ConA. 3-methyladenine (3MA) or rapamycin were used to determine the role of autophagy in FK866-conferred hepatoprotection. In primary hepatocytes, autophagy was inhibited by 3MA or autophagy-related protein 7 (*Atg7*) small interfering RNA (siRNA). JNK was suppressed by SP600125 or *Jnk* siRNA. FK866 alleviated hepatotoxicity and increased autophagy while decreased JNK activation. Suppression of autophagy abolished the FK866-conferred protection. Inhibition of JNK increased autophagy and exhibited strongly protective effect. Collectively, FK866 could ameliorate GaIN/LPS and ConA-induced ALF through induction of autophagy while suppression of JNK. These findings suggest that FK866 acts as a simple and applicable preconditioning intervention to protect against ALF; autophagy and JNK may also provide therapeutic targets for ALF treatment.

## Introduction

Acute liver failure (ALF) is a life-threatening illness wherein sudden loss of liver function leads to jaundice, coagulopathy, hepatic encephalopathy, and multiple organ failure^1^. Drugs, viruses, autoimmune factors, toxins, and shock are the prominent causes of ALF^2^. The mortality rate of ALF is as high as 40% to 50%, but thus far, only limited therapeutic approaches are available to recover liver function^2–4^. Therefore, better research on novel drugs for the prevention and treatment of ALF is of great importance.

About 100 years’ worth of literature indicates that nicotinamide adenine nucleotide (NAD^+^) functions as a cofactor or a substrate in oxidation-reduction reactions, and plays pivotal roles in the modulation of numerous cellular metabolic activities and biological and biochemical processes^5, 6^. NAD^+^ is replenished most from nicotinamide. Nicotinamide phosphoribosyltransferase (NAMPT) is the key rate-limiting enzyme involved in the NAD^+^ synthesis pathway from the natural precursor nicotinamide, but NAMPT also acts as a pro-inflammatory cytokine^7^. FK866, a highly specific small molecule noncompetitive inhibitor of NAMPT, was first recognized for its cancer therapy properties. It has been reported that FK866 manifests a wide range of antitumor activity both *in vitro* and *in vivo* ^8–11^, and exhibits a capacity to improve the sensitivity of radiation therapy in mammary carcinoma^12^. FK866 has also been observed to be protective in several inflammatory diseases such as osteoarthritis, arthritis, axonopathies, autoimmune encephalitis, and neutrophil-mediated injury in myocardial infarction^13–16^. In addition, FK866 has recently been noted for its role in the amelioration of acute hepatic injury in mice models^17^. However, the mechanism by which FK866 confers protection against hepatic injury is still largely unknown.

Autophagy is a well-conserved catabolic process characterized by the formation of autophagosomes engulfing intracellular long-lived proteins and organelles, fusing with lysosome, and degrading their contents^18^. Autophagy is necessary for maintenance of cellular homeostasis by removing misfolded large molecules and dysfunctional organelles^19^. Recent studies increasingly support that autophagy protects against hepatotoxicity induced by insults, including acetaminophen (APAP), ischemia/reperfusion, and overload of fatty acids^20–22^. Moreover, it has been reported that FK866 could induce autophagy in SH-SY5Y neuroblastoma cells and multiple myeloma cells^23, 24^. However, whether autophagy is implicated in FK866-afforded hepatoprotective function and the underlying mechanisms remain unclear.

C-jun N-terminal kinase (JNK) is an important subfamily of mitogen-activated protein kinases (MAPKs), and is crucial for cellular reaction to a variety of stimuli such as neurotransmitters, cytokines, hormones, and cell stress^25^. Several studies have convincingly documented that JNK signaling plays a critical role in determining hepatocyte damage during liver failure^26–29^. In addition, JNK signaling has been observed in the modulation of autophagy. Nevertheless, the role of JNK in autophagy is controversial. On the one hand, it is known as an essential kinase in the activation of autophagy^30^; on the other hand, JNK has been reported to be a potent negative regulator of autophagic response^31–34^.

In light of these observations, we hypothesize that FK866 could increase autophagy and subsequently ameliorate liver damage and that the JNK signaling pathway may participate in the regulation of autophagy. In the present study, we aimed to investigate the contribution of autophagy in FK866-conferred hepatoprotective effect and the regulatory mechanisms of autophagy, particularly its possible link to the JNK pathway.

## Results

### FK866 attenuates GaIN/LPS or ConA-induced ALF in mice

The serum levels of alanine aminotransferase (ALT) and aspartate aminotransferase (AST) and the liver histology were used to evaluate liver damage. The serum levels of ALT and AST were markedly higher at 3, 5, and 7 h following GaIN/LPS treatment than those in the control group (Supplementary Fig. S1a). Similarly, the serum levels of ALT and AST were significantly increased at 3 and 6 h after ConA treatment (Supplementary Fig. S1a). The liver histology confirmed the serum aminotransferase evaluation of liver damage. Severe necrosis, confluent hemorrhage, and infiltration of inflammatory cells were present in the liver tissue from the GaIN/LPS and ConA treatment groups (Supplementary Fig. S1b).

**Figure 1:**
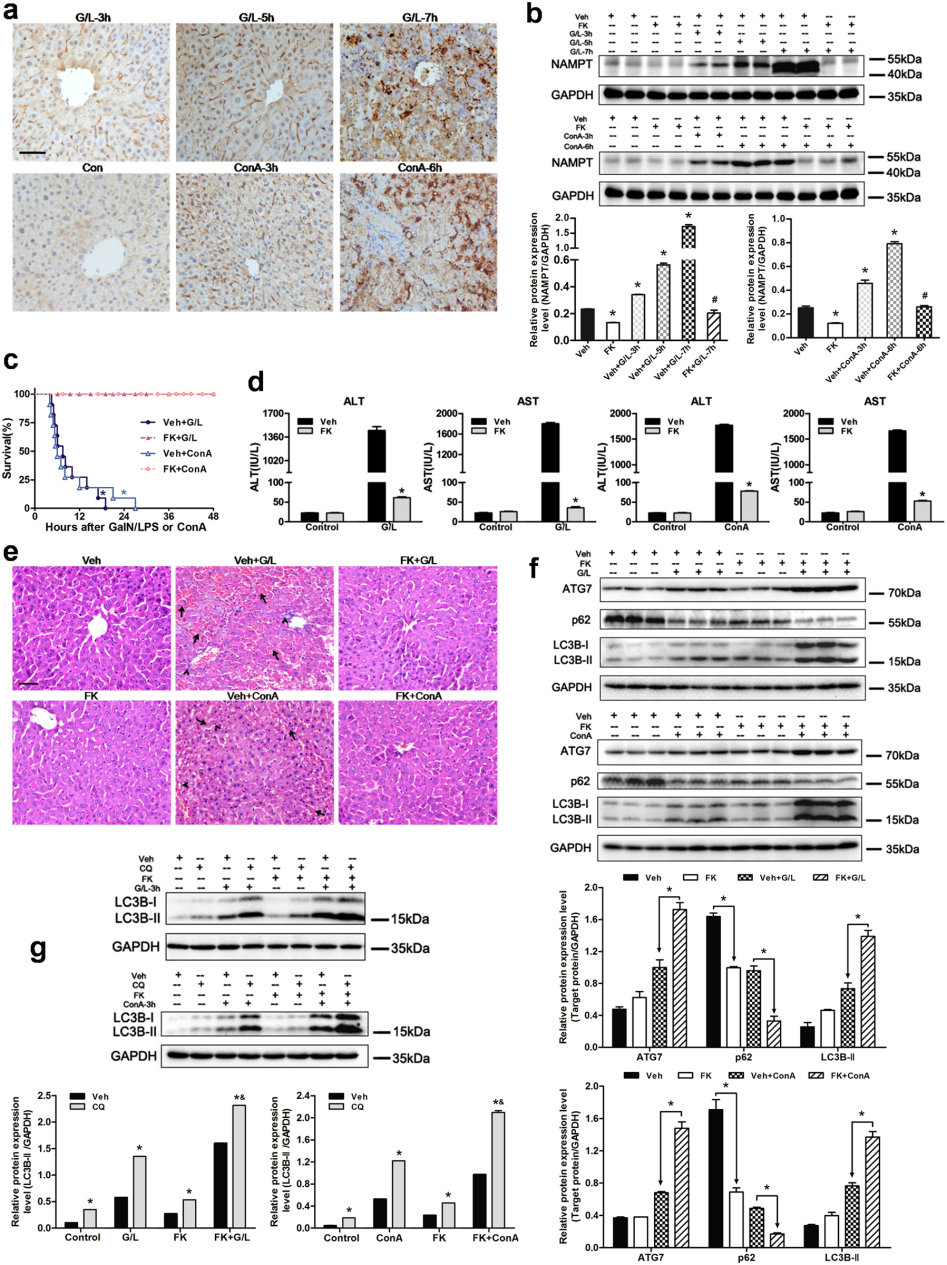
FK866 pre-treatment ameliorates GaIN/LPS- or ConA-induced ALF in mice and stimulates autophagy. Mice were subjected to GaIN/LPS (G/L, 600 mg/kg /0.5 μg/kg, IP) or ConA (20 mg/kg, IV) treatment with or without FK866 (FK, 10 mg/kg, IP), respectively. (**a**) Representative immunohistochemistry for NAMPT on paraffin-embedded liver tissue sections from control (Con) mice and G/L or ConA-induced mice. Original magnification × 400, scale bar 50 µm. n = 4 per group. (**b**) Western blot analysis of NAMPT protein expression in the presence or absence of FK. The data are shown as the means ± SEM. n = 4–6 per group. *P < 0.05 compared to the vehicle (Veh) group, ^#^ *P* < 0.05 compared to the Veh + G/L-7 h or Veh + ConA-6 h group. (**c**) The Kaplan-Meier method was used to determine the difference in survival rate after G/L or ConA challenge with or without FK. n = 11 per group. *P < 0.05 compared to the FK-treated group. (**d**) Quantification of serum ALT and AST levels. The data are shown as the means ± SEM. n = 6 per group. *P < 0.05 vs. the corresponding Veh group. (**e**) Routine histopathology was performed on formalin-fixed liver sections obtained from mice subjected to G/L or ConA challenge with or without FK. The arrows denote hepatocellular necrosis; the arrowheads denote infiltrating inflammatory cells. Original magnification × 400, scale bar 50 µm. n = 4 per group. (**f**) The protein expression levels of ATG7, p62 and LC3B were measured by western blot analysis. The data are shown as the means ± SEM. n = 4–6 per group. *P < 0.05 indicates significant differences. (**g**) To measure autophagic activity, mice received chloroquine (CQ, 60 mg/kg, 30 min, IP) prior to GaIN/LPS (G/L, 600 mg/kg /0.5 μg/kg, 3 h, IP) or ConA (20 mg/kg, 3 h, IV) challenge. Expression levels of LC3B are shown as a densitometric graph of the optical density-based data of immunoblot. The data are shown as the means ± SEM. n = 4–6 per group. *P < 0.05 vs. the corresponding Veh group, ^&^ *P* < 0.05 vs. the corresponding G/L or ConA group.

As shown in Fig. 1a, NAMPT immunohistochemistry demonstrated NAMPT expression were increased in the liver sections from mice treated with GaIN/LPS or ConA. In accordance with the immunohistochemistry data, the protein expression levels of NAMPT were increased at 3, 5, and 7 h following GaIN/LPS treatment or at 3 and 6 h after ConA treatment (Fig. 1b). To investigate the role of NAMPT in ALF, mice received three intraperitoneal injections of NAMPT-specific inhibitor FK866 at 24, 12, and 0.5 h before treatment with GaIN/LPS and ConA. Meanwhile, administration with FK866 significantly reduced the NAMPT protein expression levels (Fig. 1b). As shown in Fig. 1c, all animals died within 20 h and 30 h in the vehicle + GaIN/LPS group and the vehicle + ConA group, respectively, whereas the survival rate was 100% within 48 h both in the FK866 + GaIN/LPS group and the FK866 + ConA group. GaIN/LPS-induced ALF significantly increased serum ALT and AST levels, which drastically decreased by FK866 pretreatment. The serum levels of ALT and AST were markedly lower in the FK866-treated group, ALT from 1454 ± 54 IU/L to 61 ± 2 IU/L, and AST from 1800 ± 21 IU/L to 35 ± 3 IU/L at 7 h after GaIN/LPS treatment, respectively (*P* < 0.05). Similar results were obtained from the FK866 + ConA group versus the vehicle + ConA group (Fig. 1d). Consistent with the functional mechanism, the liver histology sections from the FK866 pretreatment groups contained less vessel congestion, reduced infiltration of inflammatory cells, and no evidence of necrosis (Fig. 1e). Furthermore, the messenger RNA (mRNA) expression levels of the proinflammatory cytokines such as tumor necrosis factor (*Tnf*)*a*, interleukin (*Il*)*1b*, and *Il6* in the FK866 pretreatment groups were significantly lower than those in the GaIN/LPS and the ConA without FK866 groups, accounting for approximately 3-fold, 10-fold, 10-fold decreases in the GaIN/LPS treatment group, and 3-fold, 10-fold, 7-fold decreases in the ConA treatment group, respectively (Supplementary Fig. S1c). To determine the effect of post-treatment with FK866, mice were injected with FK866 at 1 and 3 h after treatment with GaIN/LPS or ConA, respectively. FK866 post-treatment reduced the mortality rate of GaIN/LPS or ConA-treated mice (Supplementary Fig. S2a). In addition, FK866 post-treatment groups represented less hepatic histological damage (Supplementary Fig. S2b). Accordingly, post-treatment with FK866 significantly decreased the aminotransferase levels in mice serum, ALT from 1544 ± 32 IU/L to 101 ± 5 IU/L, or from 1793 ± 34 IU/L to 111 ± 3 IU/L at 6 h after GaIN/LPS or ConA treatment, respectively (*P* < 0.05). The AST levels represented the similar trends (Supplementary Fig. S2c). Furthermore, the mRNA expression levels of *Tnfa*, *Il1b*, and *Il6* in the FK866 post-treatment groups were lower than the corresponding vehicle treatment groups, showing about 2-fold, 5-fold, 5-fold decreases in the GaIN/LPS-treated group, and 2-fold, 5-fold, 4-fold decreases in the ConA-treated group, respectively (Supplementary Fig. S2d). These results indicate that both pretreatment and post-treatment with FK866 could attenuate the GaIN/LPS and ConA-induced ALF in mice, and the effect of pretreatment with FK866 is much better in response to GaIN/LPS or ConA challenge.

### FK866 upregulates autophagy during ALF in mice

To determine whether FK866 could induce autophagy in GaIN/LPS or ConA-induced liver injury, the expression patterns of several autophagy indicators were examined using western blot analysis. In comparison with the vehicle group, the protein expression levels of microtubule-associated light chain (LC)3B-II and autophagy-related protein 7 (ATG7) increased at 3, 5, and 7 h after treatment with GaIN/LPS, but the p62 expression decreased at corresponding times (Supplementary Fig. S1d). Likewise, similar trends were obtained in the ConA treatment groups (Supplementary Fig. S1d). These data suggest that autophagy might be an adaptive response to GaIN/LPS and ConA challenge. As shown in Fig. 1f, the ATG7 and LC3B-II levels in the livers with FK866 pretreatment were higher than those without FK866 after GaIN/LPS treatment, whereas the p62 levels were lower in the FK866 + GaIN/LPS group than those in the vehicle + GaIN/LPS group. Similar trends were found in the ConA treatment with or without FK866 groups. FK866 increased LC3B-II and ATG7 expression, but decreased p62 expression in response to GaIN/LPS or ConA challenge. LC3B is detected as two bands following western blot: one represents the cytosolic LC3B-I and the other LC3B-II, which is conjugated with phosphatidylethanolamine and is a component of the autophagosome membrane^35^. The expression level of LC3B-II is closely proportional to the autophagosome number and is consequently used as a good indicator for autophagosome presence^35, 36^. The LC3B-II level can be enhanced by activation of autophagy, and inhibition of the autophagic maturation process during which autophagosomes fuse with lysosomes could also lead to an increase of LC3B-II and autophagosomes. To clarify whether the observed LC3B-II accumulation in mice liver tissue reflected an active autophagy rather than a defect in autophagosome-lysosome fusion, mice were treated with chloroquine (CQ), a specific lysosomotropic reagent that blocks lysosomal acidification and the fusion of autophagosomes and lysosomes^37^. As shown in Fig. 1g, FK866 pretreatment demonstrated an increase in LC3B-II expression levels, and the administration of CQ led to further significant accumulation of LC3B-II in mice liver tissue, demonstrating that LC3B-II accumulation was not due to impaired autophagic maturation step. Taken together, these findings confirmed that FK866 could enhance hepatic autophagic activity in ALF mice models.

### FK866 ameliorates ALF in mice through upregulation of autophagy

**Figure 2:**
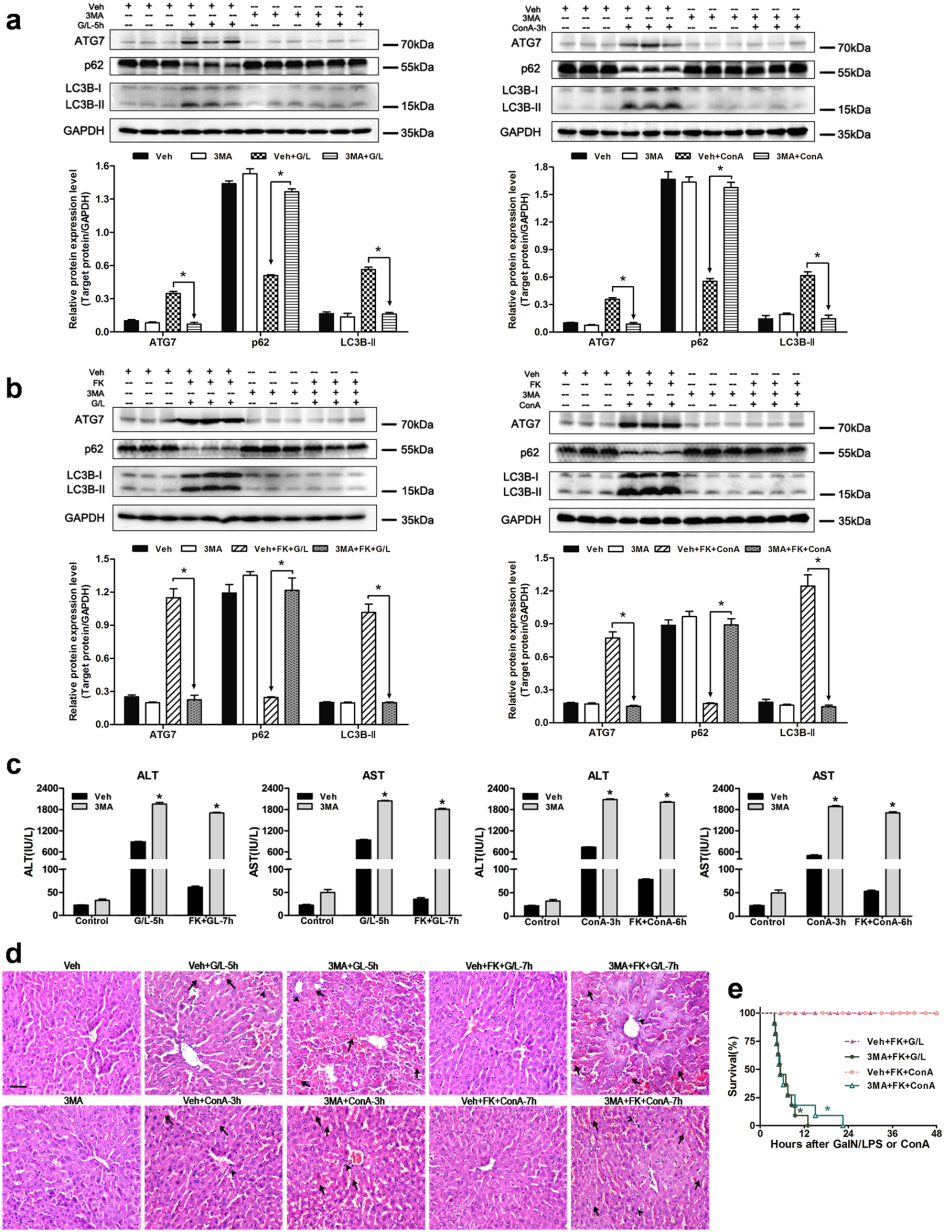
Autophagy inhibition by 3MA abolishes the FK866-conferred hepatoprotective effect in mice. Mice were injected with 3MA (30 mg/kg, 30 min, IP) prior to GaIN/LPS (G/L, 600 mg/kg /0.5 μg/kg, IP) or ConA (20 mg/kg, IV) challenge in the presence or absence of FK866 (FK, 10 mg/kg, IP). (**a**) Measurement of autophagy indicators in mice liver. The data are shown as the means ± SEM. n = 4–6 per group. *P < 0.05 indicates significant differences. (**b**) Western blot analysis of ATG7, p62, and LC3B protein expression in the presence or absence of 3MA. The data are shown as the means ± SEM. n = 4–6 per group. *P < 0.05. (**c**) Determination of serum ALT and AST levels. The data are shown as the means ± SEM. n = 6 per group. *P < 0.05 vs. the corresponding vehicle (Veh) group. (**d**) Representative images of liver injury. The arrows denote hepatocellular necrosis; the arrowheads denote infiltrating inflammatory cells. Original magnification × 400, scale bar 50 µm. n = 4 per group. (**e**) Survival rate of mice after G/L or ConA with FK in the presence or absence of 3MA. n = 11 per group. *P < 0.05 compared to the corresponding Veh-treated group.

To determine the contribution of autophagy to FK866-conferred benefit in ALF, mice were pretreated with 3-methyladenine (3MA) before treatment with GaIN/LPS or ConA. 3MA, as the class-III phosphoinositide 3-kinase (PI3K) inhibitor, has been widely used to suppress autophagy. As shown in Fig. 2a, 3MA treatment decreased the protein expression levels of LC3B-II and ATG7, and the degradation of p62 at 5 h after GaIN/LPS or 3 h after ConA treatment. In addition, 3MA treatment blocked the increase in LC3B-II and ATG7, and the p62 degradation conferred by FK866 in ALF mice models (Fig. 2b). Moreover, 3MA treatment exacerbated liver damage characterized by the markedly increased serum aminotransferase levels and severe hepatocellular necrosis in response to 5-h GaIN/LPS or 3-h ConA challenge (Fig. 2c,d). Furthermore, 3MA treatment abolished the FK866-mediated decrease in serum aminotransferase levels, the hepatocellular damage, and the mortality rate (Fig. 2c–e). These results suggest that FK866 plays a protective role in GaIN/LPS and ConA-induced ALF through the induction of autophagy.

### Induction of autophagy by rapamycin reduces liver injury in mice

**Figure 3:**
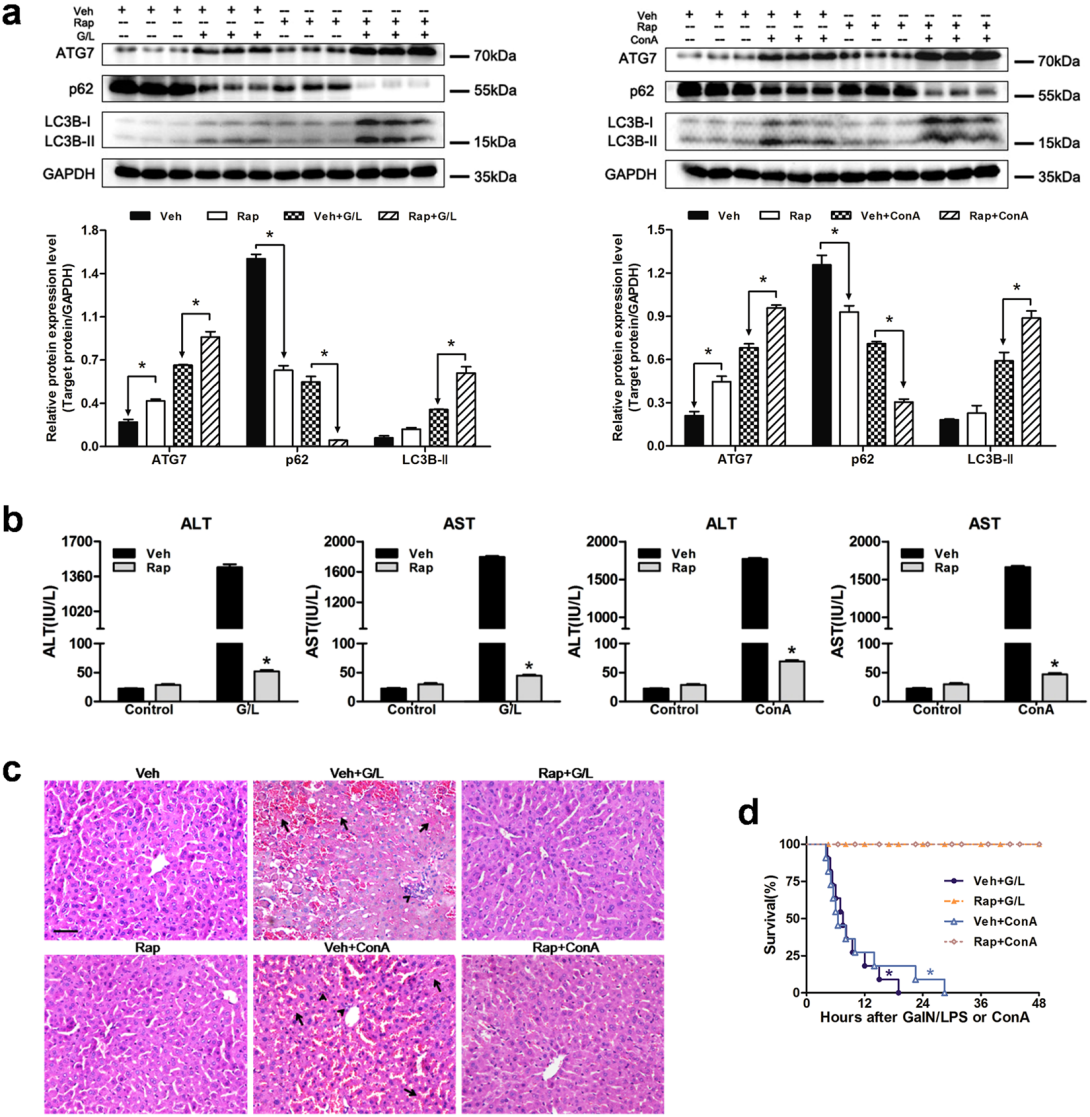
Autophagy induction by rapamycin attenuates ALF caused by GaIN/LPS or ConA in mice. Mice were pretreated with rapamycin (Rap, 2 mg/kg, 30 min, IP) prior to GaIN/LPS (G/L, 600 mg/kg /0.5 μg/kg, 7 h, IP) or ConA (20 mg/kg, 6 h, IV) challenge. (**a**) Determination of ATG7, p62, and LC3B expression in the presence or absence of Rap. The data are shown as the means ± SEM. n = 4–6 per group. *P < 0.05 indicates significant differences. (**b**) Quantification of serum aminotransferase levels. The data are shown as the means ± SEM. n = 6 per group. *P < 0.05 vs. the corresponding vehicle (Veh) group. (**c**) H&E staining of mice livers. The arrows denote hepatocellular necrosis; the arrowheads denote infiltrating inflammatory cells. Original magnification × 400, scale bar 50 µm. n = 4 per group. (**d**) Pretreatment with Rap increased survival rate of mice subjected to G/L or ConA challenge. n = 10–11 per group. *P < 0.05 compared to the Rap-treated group.

To further elucidate whether the activation of autophagy could mimic the protective effects afforded by FK866, mice were injected with rapamycin prior to the treatment of GaIN/LPS and ConA. The western blot results demonstrated that rapamycin pretreatment induced autophagy, as indicated by increase in LC3B-II, ATG7 expression and degradation of p62 (Fig. 3a). Rapamycin pretreatment reduced the elevation of the serum ALT and AST levels in response to GaIN/LPS and ConA treatment (Fig. 3b). A histological analysis showed minor damage in the liver tissues obtained from rapamycin-treated mice (Fig. 3c). Accordingly, the survival rate of GaIN/LPS and ConA-treated mice increased up to 100% within 48 h in the presence of rapamycin (Fig. 3d). Therefore, the activation of autophagy protected against GaIN/LPS and ConA-induced liver failure, supporting the concept that autophagy plays a critical role in the FK866-afforded protection.

### FK866-induced autophagy is dependent on JNK signaling

**Figure 4:**
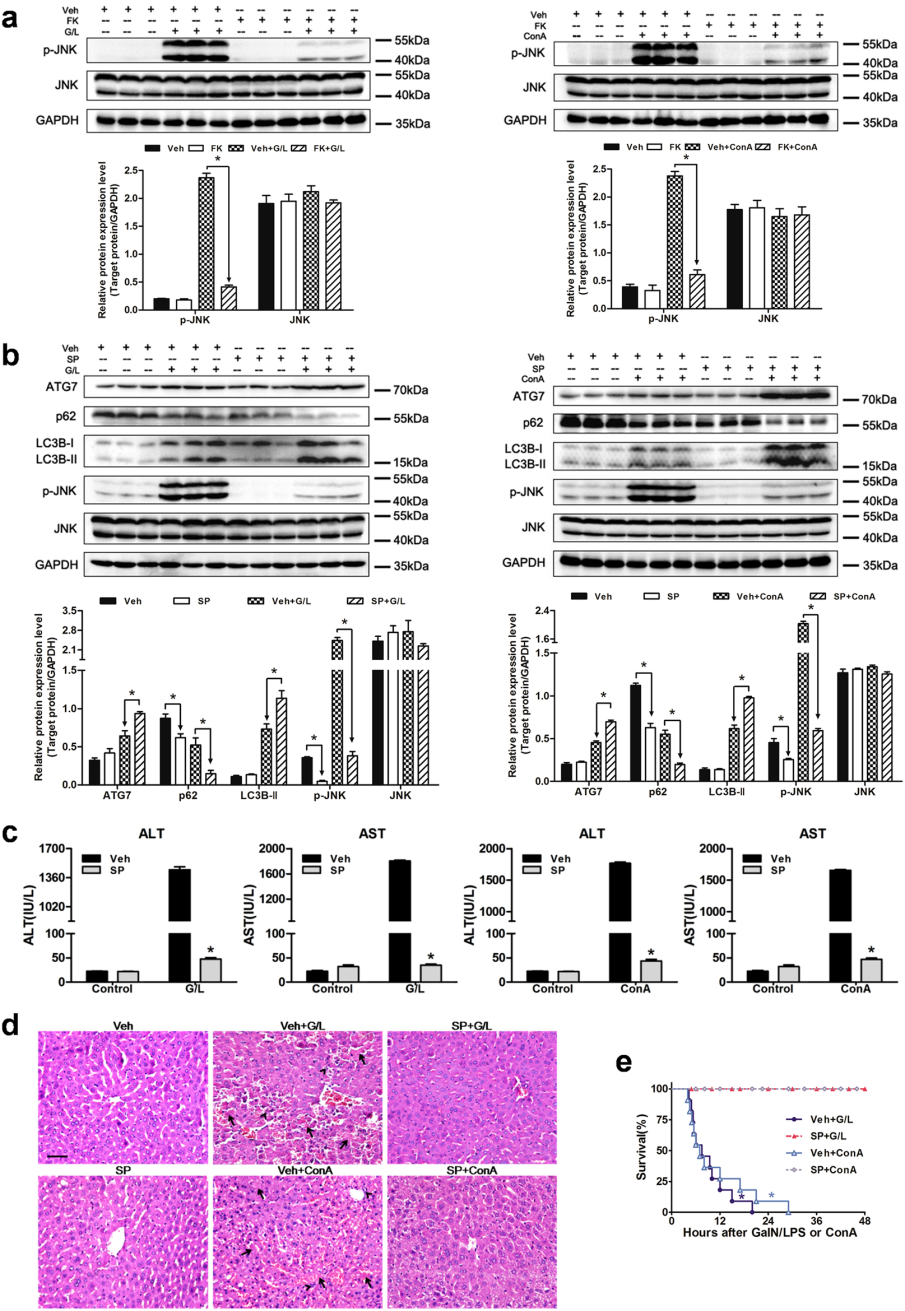
FK866-induced autophagy is dependent on JNK signaling in mice. Mice were injected with SP600125 (SP, 15 mg/kg, 30 min, IP) prior to GaIN/LPS(G/L, 600 mg/kg /0.5 μg/kg, 7 h, IP) or ConA (20 mg/kg, 6 h, IV) challenge. (**a**) Measurement of JNK activity with or without FK. The data are shown as the means ± SEM. n = 4–6 per group. *P < 0.05 indicates significant differences. (**b**) The protein expression levels of p-JNK, JNK, and autophagy indicators are shown as densitometric graphs of the optical density-based data of immunoblot. The data are shown as the means ± SEM. n = 4–6 per group.*P < 0.05. (**c**) Evaluation of serum ALT and AST levels. The data are shown as the means ± SEM. n = 6 per group. *P < 0.05 vs. the corresponding vehicle (Veh) group. (**d**) Representative images of liver histology. The arrows denote hepatocellular necrosis; the arrowheads denote infiltrating inflammatory cells. Original magnification × 400, scale bar 50 µm. n = 4 per group. (**e**) Pretreatment with SP increased survival rate of mice subjected to G/L or ConA insult. n = 10–11 per group. *P < 0.05 compared to the SP-treated group.

It has been reported that the JNK signaling pathway is implicated in the modulation of autophagic response^31–34^. To investigate the role of JNK in the FK866-induced autophagy, total JNK and phosphorylated JNK (p-JNK) protein expression were determined first in the presence or absence of FK866. As shown in Fig. 4a, the total JNK expression did not differ among groups, whereas the p-JNK expression was dramatically stronger in the vehicle + GaIN/LPS and vehicle + ConA groups than that in the FK866 pretreatment group. JNK activity was then suppressed using a small molecular inhibitor SP600125 prior to the treatment of GaIN/LPS and ConA. Interestingly, treatment with SP600125 decreased the activation of JNK and increased autophagy as measured by higher LC3B-II and ATG7 levels and lower p62 expression levels (Fig. 4b). Furthermore, the inhibition of JNK activation by SP600125 ameliorated GaIN/LPS and ConA-induced liver damage, as represented by lower serum aminotransferase levels and less hepatic histological injury as well as higher survival rates in the SP600125 pretreatment groups (Fig. 4c–e).

### FK866 alleviates hepatotoxicity through increasing autophagy *in vitro*

**Figure 5:**
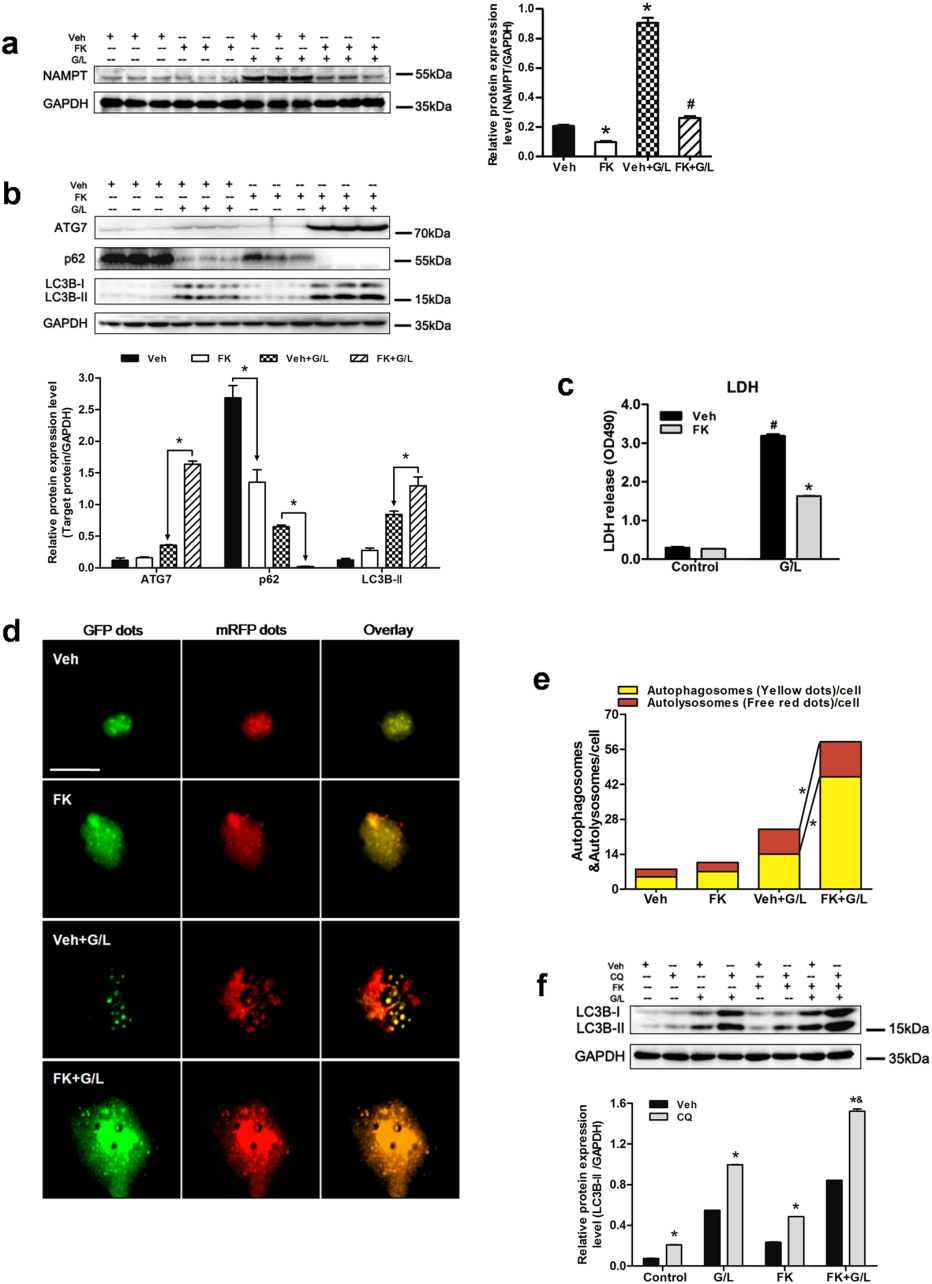
FK866 induces autophagy and alleviates hepatotoxicity induced by GaIN/LPS in primary hepatocytes. Hepatocytes were pretreated with either FK866 (FK, 100 nM, 30 min) or vehicle (Veh) and followed by GaIN/LPS (G/L, 1 mg/mL /30 ng/mL, 24 h) challenge. (**a**) Western blot analysis of NAMPT protein expression in the presence and absence of FK. The data are shown as the means ± SEM of three independent experiments performed in duplicate. *P < 0.05 compared to the Veh group, ^#^ *P* < 0.05 compared to the Veh + G/L group. (**b**) Measurement of ATG7, p62, and LC3B expression in the presence or absence of FK. The data are shown as the means ± SEM of three independent experiments. *P < 0.05 indicates significant differences. (**c**) Examination of LDH release of primary hepatocytes. The data are shown as the means ± SEM of three independent experiments. *P < 0.05 vs. corresponding Veh group, ^#^ *P* < 0.05 vs. the corresponding control group. (**d**) Representative fluorescence micrographs showed autophagy vacuoles in hepatocytes with G/L in the presence or absence of FK from a pool of at least 10 images. Original magnification × 200, scale bar 50 µm. (**e**) Quantification of autophagosomes and autolysosomes. *P < 0.05. (**f**) Western blot analysis of LC3B protein expression in the presence and absence of chloroquine (CQ). The data are shown as the means ± SEM of three independent experiments. *P < 0.05 vs. the corresponding Veh group, ^&^ *P* < 0.05 vs. the CQ + G/L group.

In agreement with the animal model studies, the protein expression levels of NAMPT were upregulated in response to GaIN/LPS challenge, but administration with FK866 significantly downregulated the NAMPT levels in hepatocytes (Fig. 5a). In addition, pretreatment with FK866 enhanced the expression of LC3B-II, ATG7, and the degradation of p62 compared to that in the GaIN/LPS treatment without FK866 group *in vitro* (Fig. 5b). Accordingly, FK866 pretreatment also resulted in a reduced cell death induced by GaIN/LPS, as measured by relative LDH levels (Fig. 5c). To further observe the formation of autophagosomes and autolysosomes in the process of autophagy, primary mouse hepatocytes were infected with adenovirus-encoding mRFP-GFP-LC3. The GFP signal is susceptible to the acidic environment of lysosomes where its fluorescence disappears, whereas mRFP is more stable and maintains its fluorescence. Thus, co-localization of both GFP and mRFP puncta that appear yellow is indicative of autophagosomes; the free mRFP puncta without GFP corresponds to autolysosomes^38^. In the present study, the numbers of green and red dots were slightly increased after treatment with GaIN/LPS (Fig. 5d). Besides, more yellow and red dots were seen in the FK866 + GaIN/LPS group, indicating FK866 could stimulate autophagy in hepatocytes (Fig. 5d,e). As shown in Fig. 5f, primary hepatocytes pretreated with FK866 exhibited a significant increase in autophagic activity in contrast to the hepatocytes without FK866 following GaIN/LPS insult.

**Figure 6:**
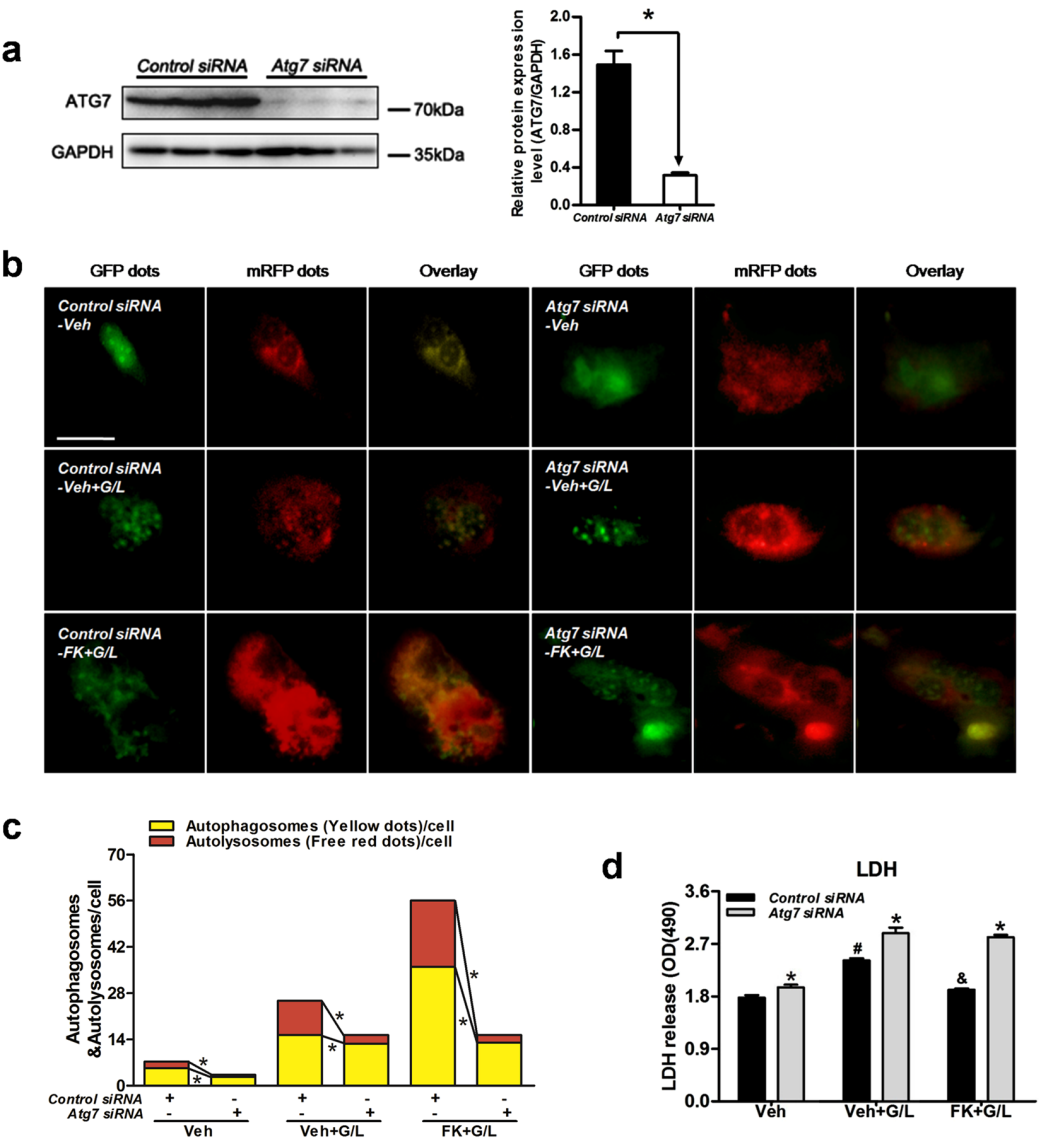
Knockdown of *Atg7* abolishes FK866-conferred hepatoprotection *in vitro*. Hepatocytes were treated with either *Control* siRNA or *Atg7* siRNA (50 nmol/L, 48 h) prior to FK866 treatment (FK, 100 nmol/L, 30 min) and followed by GaIN/LPS (G/L, 1 /mg/mL /30 ng/mL, 24 h) challenge. (**a**) ATG7 protein expression was examined by western blot analysis. The data are shown as the means ± SEM of three independent experiments performed in duplicate. *P < 0.05 indicates significant differences. (**b**) Representative fluorescence micrographs showed autophagy vacuoles in hepatocytes with or without *Atg7* siRNA from a pool of at least 10 images. Original magnification × 200, scale bar 50 µm. (**c**) Quantification of autophagosomes and autolysosomes. *P < 0.05. (**d**) Determination of LDH release of primary hepatocytes. The data are shown as the means ± SEM of three independent experiments. *P < 0.05 vs. the corresponding *Control* siRNA groups, ^#^ *P* < 0.05 vs. the corresponding vehicle (Veh) groups, ^&^ *P* < 0.05 vs. the corresponding Veh + G/L group.

To evaluate the contribution of autophagy in the FK866-conferred prevention of hepatotoxicity, hepatocytes were treated with 3MA prior to GaIN/LPS challenge. Consistent with the *in vivo* studies, 3MA treatment decreased the LC3B-II and ATG7 expression and the p62 degradation in response to GaIN/LPS in hepatocytes (Supplementary Fig. S3a). In addition, 3MA treatment significantly attenuated the increase in LC3B-II and ATG7 expression and the degradation of p62 induced by FK866 following GaIN/LPS challenge (Supplementary Fig. S3b). Treatment with 3MA also attenuated the FK866-afforded accumulation of yellow and red puncta (Supplementary Fig. S3c,d). Moreover, 3MA abolished the FK866-induced protection against hepatotoxicity (Supplementary Fig. S3e). To further determine the contribution of autophagy in FK866-conferred protective effect, autophagy was inhibited by siRNA specific for *Atg7*. As shown in Fig. 6, hepatocytes transfected with *Atg7* siRNA reduced the FK866-mediated increase in yellow and free red puncta. Accordingly, the suppression of autophagy by *Atg7* siRNA aggravated hepatotoxicity and prevented the FK866-afforded protection against cell death (Fig. 6d).

To further explore whether an autophagy inducer could mimic the hepatoprotection afforded by FK866 *in vitro*, hepatocytes were treated with rapamycin prior to GaIN/LPS challenge. Rapamycin induced autophagy in primary hepatocytes, as represented by an increased accumulation of yellow and red fluorescent dots, elevation in LC3B-II and ATG7 expression levels, and decrease in p62 expression levels in the rapamycin + GaIN/LPS group (Supplementary Fig. S4a–c). Induction of autophagy by rapamycin also attenuated hepatotoxicity caused by GaIN/LPS *in vitro* (Supplementary Fig. S4d).

### FK866-induced autophagy is associated with JNK *in vitro*

**Figure 7:**
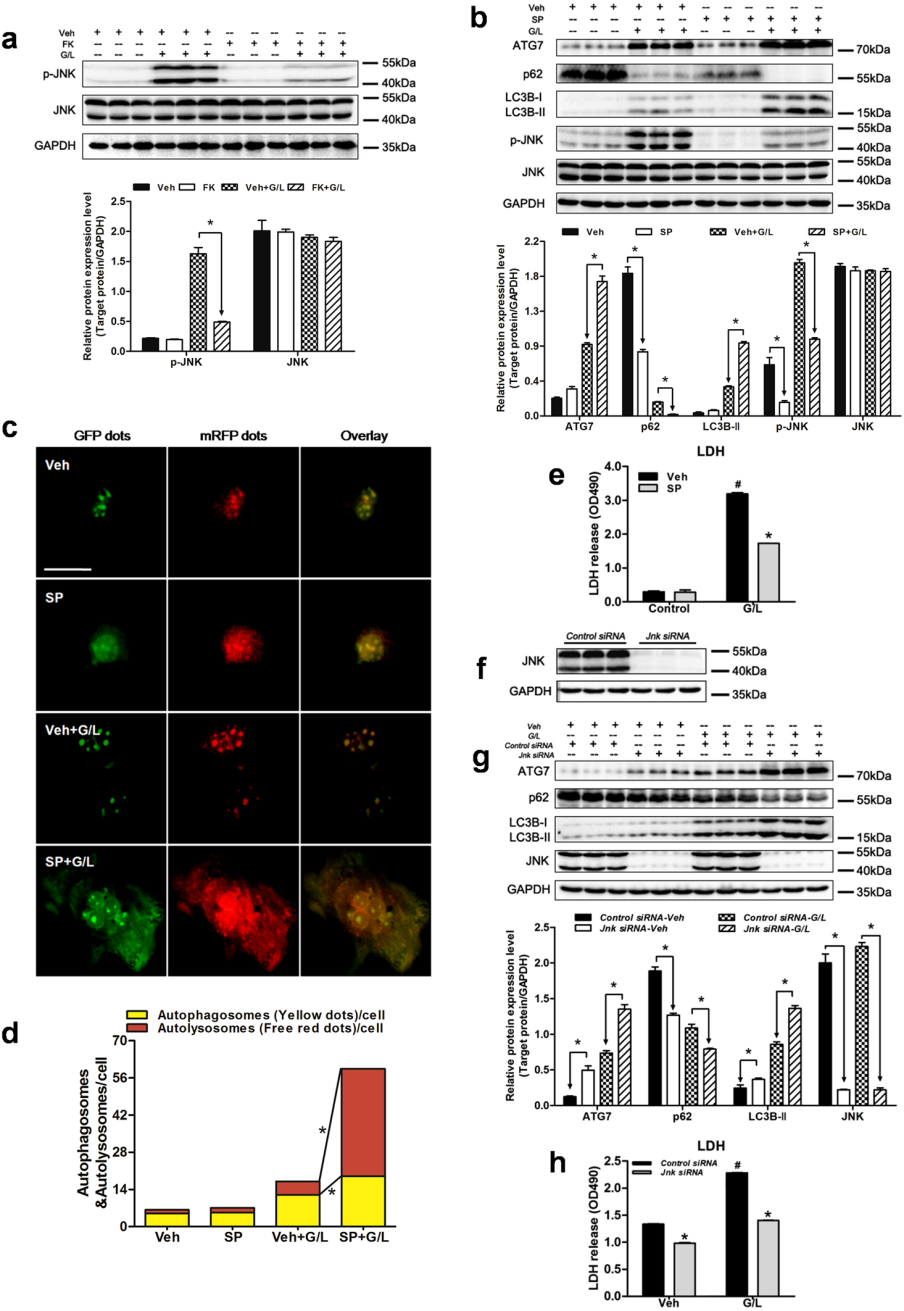
FK866-induced autophagy is associated with JNK signaling in primary hepatocytes. Hepatocytes were treated with SP600125 (SP, 20 µmol/L, 30 min) or transfected with *Jnk* siRNA (100 nmol/L, 48 h) prior to GaIN/LPS (G/L, 1 mg/mL /30 ng/mL, 24 h) challenge. (**a**) JNK activity was examined by western blot. The data are shown as the means ± SEM of three independent experiments performed in duplicate. *P < 0.05 indicates significant differences. (**b**) The protein expression levels of p-JNK, JNK, and autophagy indicators are shown as densitometric graphs of the optical density-based data of immunoblot. The data are shown as the means ± SEM of three independent experiments. *P < 0.05. (**c**) Representative fluorescence micrographs showed autophagy vacuoles in hepatocytes with G/L in the presence of SP or vehicle (Veh) from a pool of at least 10 images. Original magnification × 200, scale bar 50 µm. (**d**) Quantification of autophagosomes and autolysosomes. *P < 0.05. (**e**) Measurement of LDH release of primary hepatocytes. The data are shown as the means ± SEM of three independent experiments. *P < 0.05 vs. the corresponding Veh group. ^#^ *P* < 0.05 vs. the corresponding control group. (**f**) JNK protein expression was effectively suppressed using *Jnk*-specific siRNA in hepatocytes. (**g**) Western blot analysis of JNK, ATG7, p62, and LC3B expression in the presence or absence of *Jnk* siRNA. The data are shown as the means ± SEM of three independent experiments. *P < 0.05. (**h**) Determination of LDH levels in the cultured medium. The data are shown as the means ± SEM of three independent experiments. *P < 0.05 vs. the corresponding *Control* siRNA group. ^#^ *P* < 0.05 vs. the corresponding Veh group.

To further investigate the regulatory effect of JNK on autophagy in cultured primary hepatocytes, JNK activity was inhibited using SP600125 or siRNA specific for *Jnk*. FK866 decreased the phosphorylation of JNK in response to GaIN/LPS challenge (Fig. 7a). Treatment with SP600125 suppressed the activation of JNK, but significantly upregulated the LC3B-II and ATG7 levels, attenuated the p62 expression, and enhanced both yellow and red puncta following GaIN/LPS challenge (Fig. 7b–d). Moreover, the inhibition of JNK with SP600125 relieved the GaIN/LPS-induced hepatotoxicity (Fig. 7e). Next, JNK expression was effectively inhibited by *Jnk* siRNA (Fig. 7f). Similar to the function of SP600125, the suppression of JNK activity with *Jnk* siRNA markedly increased the LC3B-II and ATG7 expression and the p62 degradation, and ameliorated the cell death caused by GaIN/LPS accordingly (Fig. 7g,h). These findings indicate that JNK inactivation enhances autophagy in primary hepatocytes.

## Discussion

FK866 was recently reported to attenuate acute liver damage^17^, and the exact mechanism by which FK866 exerts this action remains poorly understood. Autophagy plays a vital role in protecting against liver injury caused by GaIN/LPS^39^. The aim of this study was to investigate whether FK866 could exert a hepatoprotective effect through JNK-dependent autophagy in GaIN/LPS and ConA-induced ALF. The major novel findings of this investigation are as follows: (1) FK866 pretreatment reduces hepatocellular injury both in GaIN/LPS and ConA-induced ALF models in mice and GaIN/LPS-induced hepatotoxicity in cultured hepatocytes; (2) the protective effect of FK866 is mediated through induction of autophagy; and (3) JNK may be a potent negative regulator of FK866-induced autophagy.

ALF is related to many complications and has a relatively high mortality rate. Effective treatment approaches for ALF are limited. Moschen *et al.* ^17^ recently demonstrated that NAMPT was strongly upregulated and NAMPT gene delivery aggravated liver disease in ConA-induced experimental hepatitis, whereas blocking NAMPT using FK866 protected mice from GaIN/LPS and ConA-induced acute liver injury. Consistent with these findings, we found that NAMPT expression was increased, and blocking NAMPT using FK866 attenuated GaIN/LPS or ConA-induced ALF as indicated by lower serum aminotransferase levels and hepatic inflammatory cytokine levels, fewer GaIN/LPS or ConA-associated liver histopathologic changes, and a higher survival rate. However, how FK866 exerts this beneficial effect in acute liver injury has not yet been elucidated.

Autophagy is an intracellular catabolic process that is indispensable for development, differentiation, survival, and homeostasis, which pathway is tightly regulated and highly inducible^40^. Furthermore, there is increasing evidence suggesting that autophagy plays a protective role in acute liver injury caused by a variety of challenges including LPS, cecal ligation and puncture (CLP), TNFα, APAP, ischemia/reperfusion, overload of fatty acids^18, 20–22^. It is possible that FK866 could protect liver injury through induction of autophagy. This view is supported by several recent studies. Billington *et al.* ^23^ recently demonstrated that FK866 induced autophagy in neuroblastoma cells. Cea *et al.* ^6^ found that FK866 triggered a marked increase in autophagy in multiple myeloma cells. In agreement with these observations, we demonstrated that FK866 could significantly increase LC3B-II and ATG7 expression, degrade p62, and stimulate autophagic activity both in GaIN/LPS and ConA-induced ALF models in mice and GaIN/LPS-induced hepatotoxicity in cultured hepatocytes. FK866 can also increase the formation of autophagosomes and autolysosomes in response to GaIN/LPS-induced hepatotoxicity in cultured hepatocytes. These data indicate that FK866 could enhance autophagy in GaIN/LPS and ConA-induced ALF.

To determine the involvement of an autophagic mechanism in the protection offered by FK866, FK866-induced autophagy was inhibited by 3MA. It was shown that inhibition of autophagy by 3MA diminished the FK866-afforded protective effect on GaIN/LPS and ConA-induced ALF in mice. This finding was confirmed by the data from *in vitro* studies, which showed that the inhibition of autophagy using 3MA or *Atg7* siRNA in primary hepatocytes abolished the FK866-conferred protection against GaIN/LPS-induced hepatotoxicity. These findings suggest that FK866 may at least partly exert its hepatoprotective effect through the promotion of autophagy. To further determine whether autophagy induction was responsible for FK866-induced protection, rapamycin, a widely used inducer of autophagy, was applied prior to GaIN/LPS and ConA challenge in mice. Pretreatment with rapamycin increased autophagy and reduced hepatocellular injury both in GaIN/LPS and ConA-mediated ALF in mice. Rapamycin pretreatment was associated with a remarkable protection of hepatocytes in GaIN/LPS-induced hepatotoxicity. These results show that rapamycin could mimic the FK866-afforded hepatoprotective effect. These results strongly support the concept that the protective role of FK866 may be partially through induction of autophagy.

The regulatory mechanisms of autophagy during ALF remain unclear. It is reported that JNK signaling may participate in the modulation of autophagy^41^. JNK is a family of structurally similar serine/threonine kinase, and its activity is merely detectable in the normal liver but highly inducible when cells are under stress^42^. Saberi *et al.* ^43^ reported that p-JNK was essential for APAP-induced liver injury. Moreover, Wang *et al.* ^44^ indicated that JNK over-activation was responsible for GaIN/LPS-induced injury. In addition, An *et al.* ^45^ suggested that JNK exerted a crucial role during ConA and GaIN/LPS-caused liver damage. Consistent with these observations, we demonstrated that p-JNK expression was increased and inhibition of JNK reduced liver injury in GaIN/LPS and ConA-induced ALF models in mice. Xu *et al.* ^31^ recently found that JNK inhibited autophagy in neurons by suppressing the expression of autophagy-related genes including ATG8/LC3B-II and ATG12. In addition, Basu *et al.* ^32^ demonstrated that the inactivation of JNK induced autophagy in the ocular lens by suppressing the MTOR (mechanistic target of rapamycin)-RPTOR (regulatory associated protein of MTOR, complex 1) signaling axis. In agreement with these observations, we found that FK866 decreased the p-JNK expression and activated autophagy in GaIN/LPS and ConA-induced ALF models in mice. More importantly, suppression of JNK by SP600125 increased autophagy and attenuated hepatocellular damage in mice. A similar finding was observed *in vitro*. Inhibition of JNK by SP600125 or *Jnk* siRNA increased autophagy and reduced GaIN/LPS-induced hepatotoxicity in primary hepatocytes. These results indicate that JNK may be a potent negative regulator of FK866-induced autophagy. However, the underlying mechanism of how FK866 suppresses JNK is still not well defined. NAD is an essential coenzyme of adenosine triphosphate (ATP)-synthesizing mitochondrial electron transport chain reactions and its rescue pathway is regulated by NAMPT^46^. Nahimana *et al.* ^10^ showed that FK866 led to a delayed ATP reduction after 24 h in chronic lymphocytic leukemia cells. Pittelli *et al.* ^47^ reported that exposure to FK866 prompted NAD depletion with a concomitant increase in ATP in both primary cell cultures of different species and tissues. Shang *et al.* ^48^ demonstrated that reduced ATP levels caused the activation of JNK and then promoted GaIN/LPS-induced cell death. Based on these observations, it is possible that the potent NAMPT inhibitor FK866 may modulate ATP levels and further influence JNK activity.

In conclusion, we demonstrated that FK866 could ameliorate GaIN/LPS and ConA-induced liver injury, and the protective mechanism may involve its ability to induce autophagy through the suppression of JNK. These findings may provide new insights into the functional mechanism of FK866 and an important framework for developing novel targeted clinical therapies for acute liver failure.

## Materials and Methods

### Animals and Acute Liver Failure Models

Eight-week-old male C57BL/6 mice were obtained from Wuhan University Center for Animal Experiment (Wuhan, China). All mice were bred under 12 h light/dark cycles with unlimited access to standard food and water. All mice were treated in accordance with the guidelines of the National Institutes of Health for Animal Care and Use, and the ethical approval was granted by the Tongji hospital animal ethic committee (Huazhong University of Science and Technology, Wuhan, China). GaIN/LPS- and ConA-induced ALF models were used. For the GaIN/LPS-induced ALF model, mice received intraperitoneal (IP) injections of GaIN (600 mg/kg, Sigma-Aldrich, St. Louis, MO) together with LPS (0.5 μg/kg, Sigma-Aldrich) in pyrogen-free saline, and were euthanized at 3, 5, and 7 h after GaIN/LPS treatment. Blood and livers were then collected to assess liver damage. For the ConA-induced ALF model, mice were injected intravenously (IV) with ConA (20 mg/kg, Sigma-Aldrich) in pyrogen-free saline, and were euthanized at 3 and 6 h after ConA administration. Blood and livers were then obtained. To test the potential effect of FK866 on ALF in mice, FK866 (10 mg/kg, Cayman Chemical, Michigan, USA) was administered at 24, 12, and 0.5 h prior to treatment with GaIN/LPS and ConA. To determine autophagic activity, mice were treated with chloroquine (CQ, 60 mg/kg, IP, Sigma-Aldrich). Induction or suppression of autophagy was conducted by IP injection of rapamycin (2 mg/kg, Abcam, Cambridge, UK) or 3-methyladenine (3MA, 30 mg/kg, Cayman Chemical). FK866 or rapamycin was diluted in dimethyl sulfoxide (DMSO), and 3MA was dissolved in warm saline solution. JNK activity was inhibited by SP600125 (15 mg/kg, Sigma-Aldrich).

### Determination of liver damage

To quantify the liver damage, the activities of alanine aminotransferase (ALT) and aspartate aminotransferase (AST) in serum samples were colorimetrically determined by a commercially available kit (TECO Diagnostics, Anaheim, CA) according to the manufacturer’s protocol.

### Liver Histology

The liver specimen was fixed in 4% buffered paraformaldehyde overnight at room temperature, and then dehydrated and embedded in paraffin. For histological analysis, tissue sections of 4 µm thickness were stained with hematoxylin and eosin (H&E) and evaluated by two pathologists who were not aware of sample distribution to experimental groups. Twelve fields under each condition were randomly taken at 400 × magnification.

### Immunohistochemistry (IHC)

Paraffin-embedded tissue sections were dewaxed and rehydrated. Endogenous peroxidase and non-specific binding sites were blocked immediately following the antigen retrieval, the sections were then incubated with NAMPT antibody (Santa Cruz Biotechnology, Santa Cruz, CA) diluted 1:50 in IHC antibody diluent. Peroxidase-conjugated anti-rabbit antibodies were used for secondary detection. The reaction was revealed with diaminobenzidine (DAB), and sections were counterstained with hematoxylin. Images were acquired on a microscope (Olympus, Tokyo, Japan) at a magnification of 400×.

### Primary hepatocytes culture

Primary hepatocytes were isolated from eight-week-old male C57BL/6 mice as previously described^19^. All cells were seeded into collagen-enveloped 6-well plates and were grown in Dulbecco’s modified Eagle’s medium (DMEM, Gibco BRL, Gaithersburg, MD) supplemented with 10% fetal bovine serum (Gibco), 100 U/mL penicillin, and 100 µg/mL streptomycin (Sigma) at 37 °C in an atmosphere of 5% CO_2_. After the medium was changed, the cells were stimulated with 1 mg/mL GaIN and 30 ng/mL LPS for 24 h. In other conditions, the cells were pre-incubated with FK866 (100 nmol/L) and/or CQ (10 µmol/L), 3MA (10 mmol/L), rapamycin (200 nmol/L), and SP600125 (20 µmol/L) for 30 min before stimulation. Subsequently, the cell culture supernatants and adherent cells were harvested for the experiments.

### Transient transfection of siRNA

A total of 2 × 10^5^ cells were seeded into collagen-enveloped 6-well plates and cultured for 24 h. Thereafter, *Atg7* small interfering RNA (siRNA), *Jnk* siRNA, or *Control* siRNA was transfected using Lipofectamine RNAiMAX (Invitrogen, Carlsbad, CA) following the manufacturer’s instructions. After transfection for 48 h, primary hepatocytes were treated with different conditions for further analysis.

### mRFP-GFP-LC3 adenovirus transfection

Hepatocytes were infected with adenovirus-encoding mRFP-GFP-LC3 (Hanbio, Shanghai, China) at 30 multiplicities of infection (MOIs). After transfection for 24 h, the cells were treated with GaIN and LPS in the presence or absence of FK866, 3MA, rapamycin, and SP600125 at indicated doses and durations. The cells were then fixed in 4% paraformaldehyde for 20 min at room temperature and examined under a fluorescent microscope (Olympus). The number of autophagosomes (yellow dots) and autolysosomes (red dots) per cell were calculated for the evaluation of autophagic activity.

### Cytotoxicity measurement

LDH levels in the cultured medium were determined by LDH-Cytotoxicity Colorimetric Assay Kit (Biovision, Milpitas, CA). The absorbance was measured at 490 nm using a microplate reader (BioTek, Winooski, Vermont) according to the manufacturer’s instructions.

### Protein Extraction and Western Blot Analysis

Total protein extracts were prepared from liver homogenates or primary hepatocytes using RIPA lysis buffer containing 1% Triton X-100, 0.5% sodium deoxycholate, 0.1% SDS with protease inhibitor cocktail, and phosphatase inhibitor cocktail (Roche, Mannheim, Germany). Extracts were adequately lysed on ice and centrifuged at 12,000 *g* for 15 min. Protein concentrations were determined by the BCA protein assay (Thermo Fisher Scientific, Waltham, MA). Equal amounts of protein were separated by electrophoresis through 12% SDS-PAGE gels and then transferred onto a PVDF membrane (Millipore, Darmstadt, Germany). The membranes were then blocked for 1 h at room temperature in phosphate-buffered saline containing 0.1% Tween 20 (PBST) and 5% nonfat dry milk. Subsequently, the membranes were incubated overnight at 4 °C with the primary antibodies that recognized NAMPT (Santa Cruz), LC3B (Abcam), ATG7, phosphorylated or total JNK (Cell Signaling Technology, Beverly, MA), glyceraldehyde-3-phosphate dehydrogenase (GAPDH), and p62/SQSTM1 (Sigma-Aldrich). The protein bands were detected with horseradish peroxidase-conjugated secondary antibodies (Abcam) and developed using the SuperSignal™ West Femto Maximum Sensitivity Substrate (Thermo Fisher Scientific). Western blot signals were captured by the Kodak Imaging System (Carestream Health Inc, Rochester, NY).

### Quantitative polymerase chain reaction (PCR)

Total RNA was extracted from the liver tissue using Trizol reagent (Invitrogen, Carlsbad, CA) according to the manufacturer’s protocol. PrimeScript™ first-strand kit (Takara, Otsu, Japan) was used to perform cDNA synthesis. The PCR reaction mixture was prepared using SYBR Green Real time PCR Master Mix kit (Toyobo, Osaka, Japan). The primer sequences used for amplification were as follows: *Tnfa* (GenBank accession number: U68416.1), forward primer, 5′-TGCTGGGAAGCCTAAAAGG-3′; reverse primer, 5′-CGAATTTTGAGAAGATGATCCTG-3′; *Il1b* (GenBank accession number: NM_008361.4), forward primer, 5′-TCATGGGATGATGATGATAACCTGCT-3′; reverse primer, 5′-CCCATACTTTAGGAAGACACGGATT-3′; *Il6* (GenBank accession number: M20572.1), forward primer, 5′-CACATGTTCTCTGGGAAATCGTGGA-3′; reverse primer, 5′-TCTCTCTGAAGGACTCTGGCTTTGT-3′; *Gapdh*, forward primer, 5′-TCAACAGCAACTCC CACTCTTCCA-3′; reverse primer, 5′-TTGTCATTGAGAGCAATGCCAGCC-3′. A lightcycler 480 Real time PCR System (Roche, La Jolla, CA) was used for PCR. The temperature profile was at 95 °C for 5 min, followed by 45 cycles at 95 °C for 30 s, 55 °C for 35 s, and 72 °C for 30 s.

### Statistical analysis

The data are expressed as means ± SEM. Differences between two groups were assessed by unpaired Student’s t test. Significance of differences among three or more groups was conducted with one-way analysis of variance followed by the Newman–Keuls multiple comparison test. Survival rates were evaluated by Kaplan-Meier survival analysis. A p-value below 0.05 was considered statistically significant.

## Electronic supplementary material

Supplementary information
