# Supplementary material for: FK866 attenuates acute hepatic failure through c-jun-N-terminal kinase (JNK)-dependent autophagy
Source: Sci Rep. 2017 May 19;7:2206. doi: 10.1038/s41598-017-02318-7 (PMC5438370; doi:10.1038/s41598-017-02318-7)

Supplementary information

Title:*FK866 attenuates acute hepatic failure through c-jun-N-terminal kinase (JNK)-dependent autophagy*

Authors: Enshuang Guo [1,2], Renlong Li [1,2], Jiankun Yang [3], Jun Zhang [1,2], Anyi Li [4], Yan Yang [3], Shenpei Liu [3], Anding Liu [3,*], Xiaojing Jiang [1,2,*]

Authors affiliations: [1] Graduate School, Southern Medical University, 1023 Shatai Nan Road, Guangzhou 510515, China. [2] Department of Infectious Diseases, Wuhan General Hospital, 627 Wuluo Road, Wuhan 430070, China. [3] Experimental Medicine Center, Tongji Hospital, Tongji Medical College, Huazhong University of Science and Technology, 1095 Jiefang Avenue, Wuhan 430030,China. [4]Animal Experiment Center, Tongji Hospital, Tongji Medical College, Huazhong University of Science and Technology, 1095 Jiefang Avenue, Wuhan 430030, China

***Corresponding authors:** Anding Liu, e-mail: Anding.liu@uk-essen.de; Xiaojing Jiang, e-mail: xjjiang2003@163.com

Supplementary information includes:

1. Supplementary figures
2. Supplementary figure legends
3. Original images of cropped blots

Supplementary Fig. S1

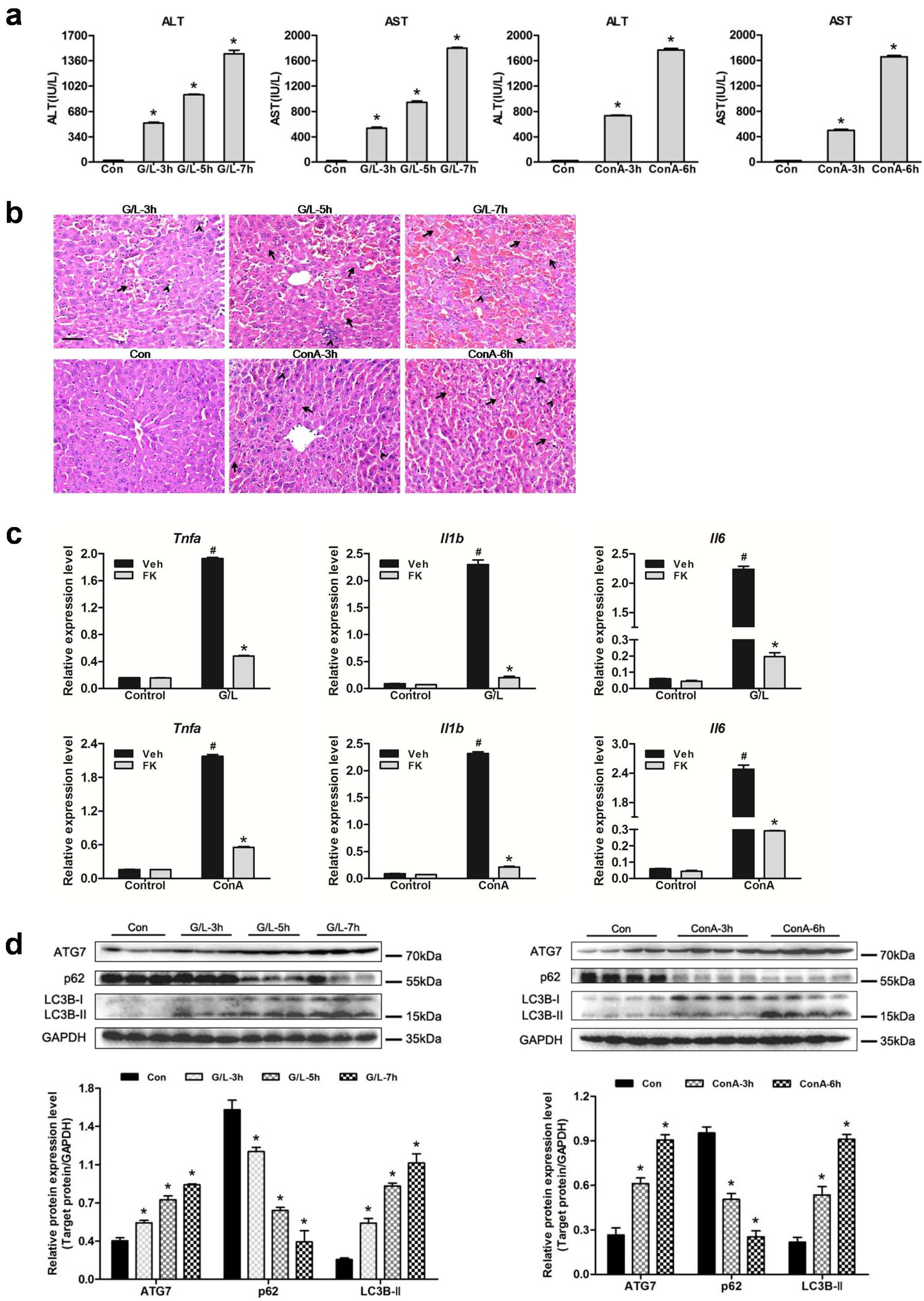

Supplementary Fig. S2

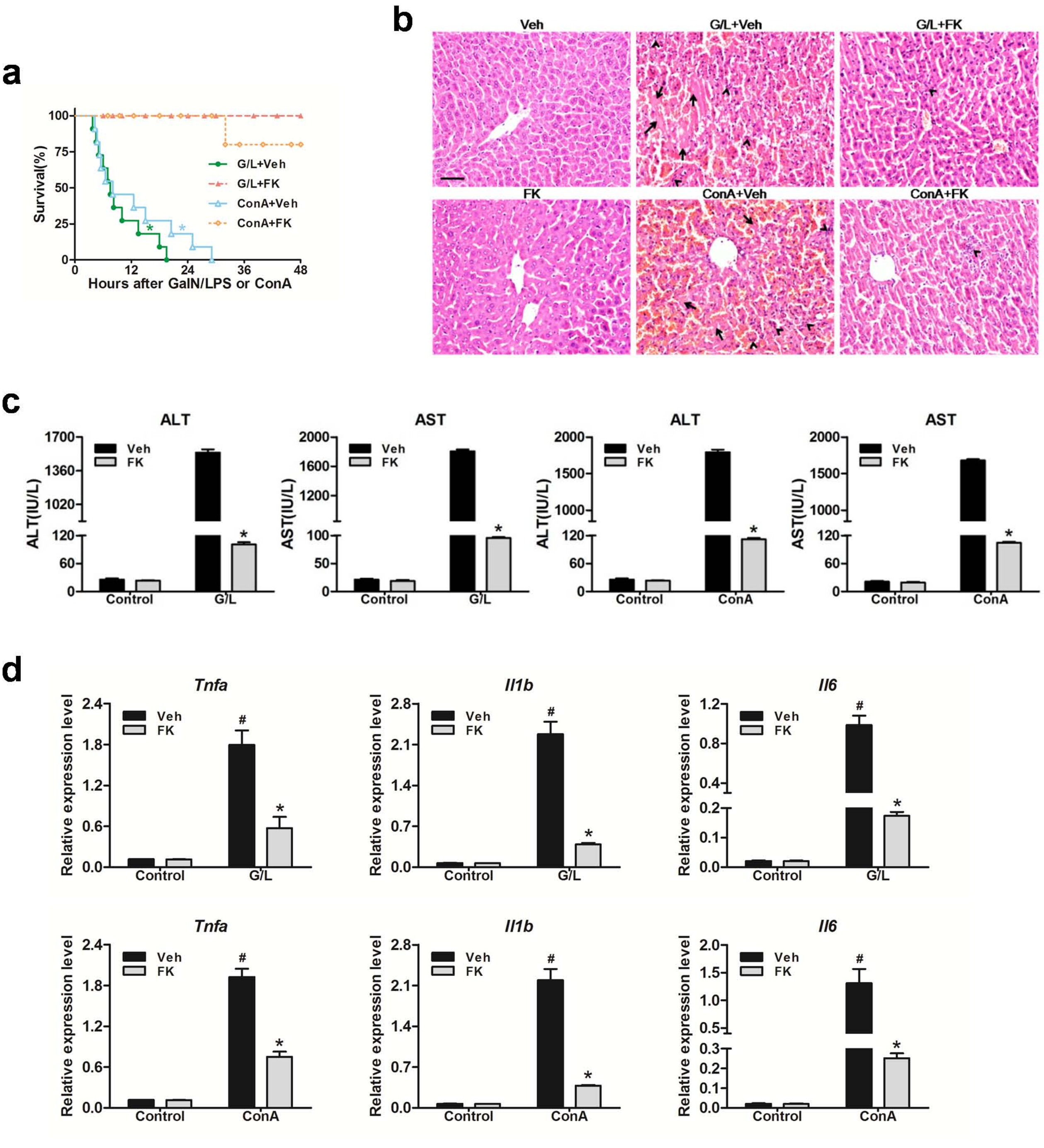

Supplementary Fig. S3

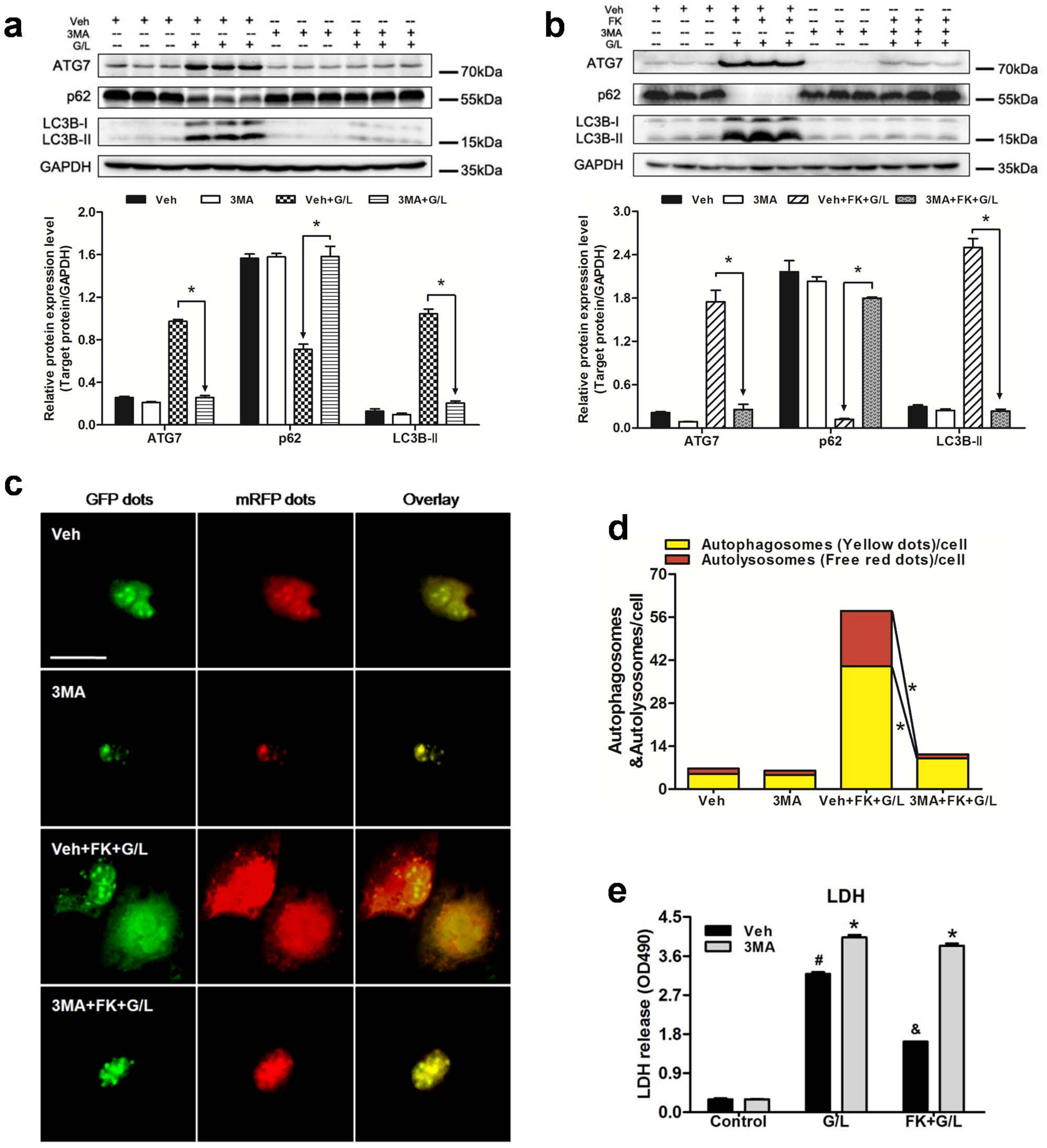

Supplementary Fig. S4

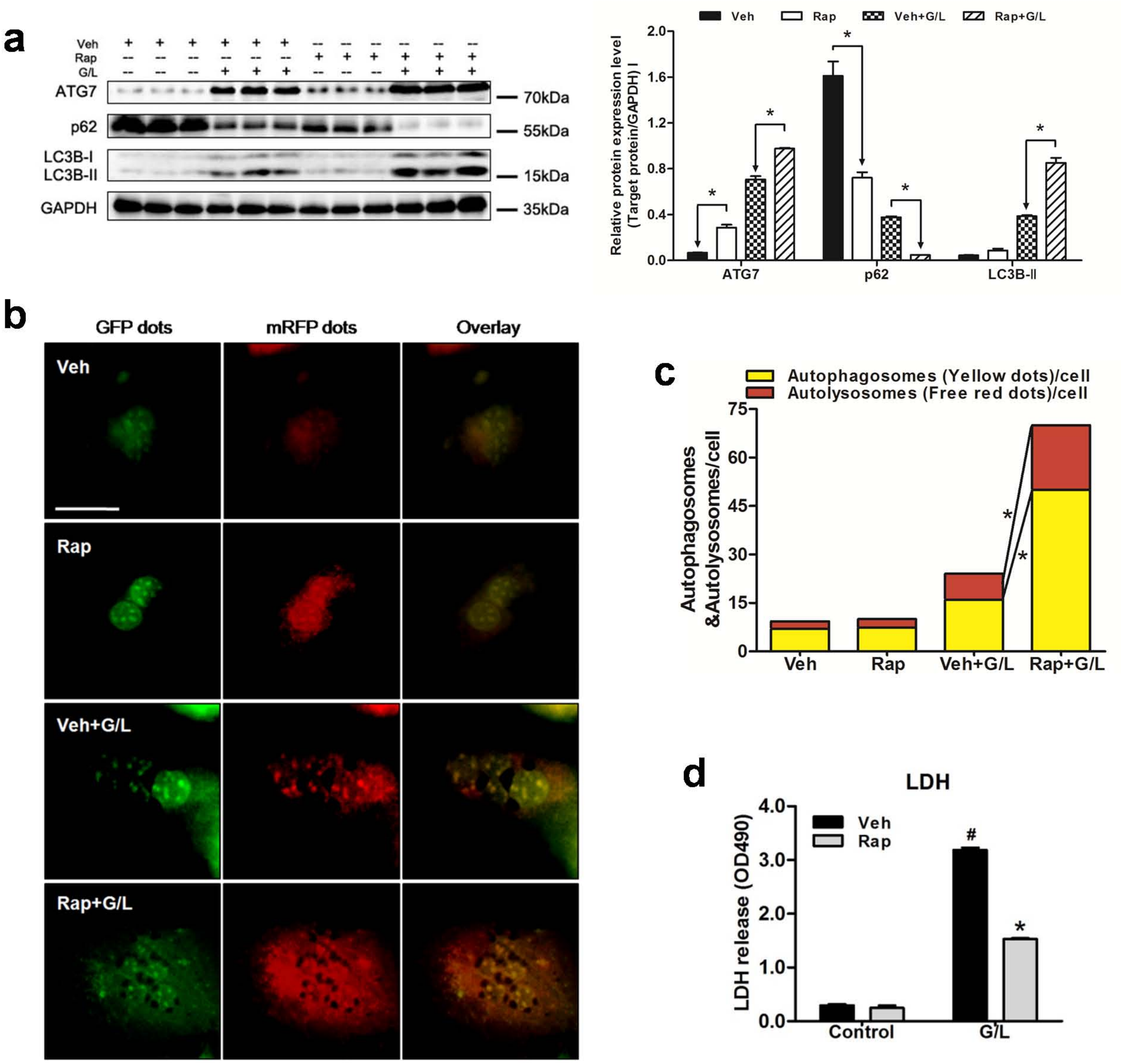

Supplementary Fig. S1. ALF is induced by GaIN/LPS or ConA. Mice were treated with GaIN/LPS (G/L, 600 mg/kg /0.5 μg/kg, IP) or ConA (20 mg/kg, IV) to induce ALF. (a) Quantification of serum ALT and AST levels. The data are shown as the means ± SEM. n = 6 per group. *$P < 0.05$ compared to the control (Con) group. (b) Representative images of liver histology. The arrows denote hepatocellular necrosis; the arrowheads denote infiltrating inflammatory cells. Original magnification ×400, scale bar 50 µm. n = 4 per group. (c) The mRNA expression levels of *Tnfa, Il1b* and *Il6* were measured by PCR. The data are shown as the means ± SEM. n = 6 per group. *$P < 0.05$ vs. the corresponding Veh group. $^{\#}P < 0.05$ vs. the corresponding control group. (d) Measurement of ATG7, p62, and LC3B expression at different time points during treatment. The data are shown as the means ± SEM. n = 4-6 per group. *$P <$ 0.05 compared to the Con-treated group.

Supplementary Fig. S2. FK866 post-treatment attenuates GaIN/LPS- or ConA-induced ALF in mice. Mice were injected with FK866 (FK, 10 mg/kg, IP) at 1 and 3 h after treatment with GaIN/LPS (G/L, 600 mg/kg /0.5 μg/kg, IP) or ConA (20 mg/kg, IV), respectively. (a) Post-treatment with FK increased survival rate of mice subjected to G/L or ConA challenge. n = 11 per group. *$P < 0.05$ compared to the FK-treated group. (b) Routine histopathology was performed on formalin-fixed liver sections obtained from mice subjected to 6 h-G/L or ConA challenge with or without FK post-treatment. The arrows denote hepatocellular necrosis; the arrowheads denote infiltrating inflammatory cells. Original magnification ×400, scale bar 50 µm. n = 4 per group. (c) The ALT and AST levels in mice serum were measured. The data are

shown as the means ± SEM. n = 6 per group. **P* < 0.05 vs. the corresponding vehicle (Veh) group. (d) The mRNA expression levels of *Tnfa*, *Il1b* and *Il6* in the mice livers. The data are shown as the means ± SEM. n = 6 per group. **P* < 0.05 vs. the corresponding Veh group. $^{\#}P < 0.05$ vs. the corresponding control group.

Supplementary Fig. S3. Inhibition of autophagy by 3MA reverses FK866-conferred hepatoprotection in vitro. Hepatocytes were treated with 3MA (10 mmol/L, 30 min) following GaIN/LPS (G/L, 1 mg/mL /30 ng/mL, 24 h) challenge with or without FK866 (FK, 100 nmol/L). (a) Determination of ATG7, p62, and LC3B expression in primary hepatocytes. The data are shown as the means ± SEM of three independent experiments performed in duplicate. **P* < 0.05 indicates significant differences. (b) Western blot analysis of autophagy indicator in the presence or absence of 3MA. The data are shown as the means ± SEM of three independent experiments. **P* < 0.05. (c) Representative fluorescence micrographs showed autophagy vacuoles in hepatocytes with FK in the presence or absence of 3MA from a pool of at least 10 images. Original magnification ×200, scale bar 50 µm. (d) Quantification of autophagosomes and autolysosomes. **P* < 0.05. (e) Measurement of LDH release of primary hepatocytes. The data are shown as the means ± SEM of three independent experiments. **P* < 0.05 vs. the corresponding vehicle (Veh) groups, $^{\#}P < 0.05$ vs. the corresponding control groups, $^{\&}P < 0.05$ vs. the corresponding G/L group.

Supplementary Fig. S4. Induction of autophagy by rapamycin reduces GaIN/LPS-induced hepatotoxicity in vitro. Hepatocytes were treated with

rapamycin (Rap, 200 nmol/L, 30 min) prior to GaIN/LPS (G/L, 1 mg/mL /30 ng/mL, 24 h) challenge. (a) The protein expression levels of ATG7, p62 and LC3B were measured by western blot analysis. The data are shown as the means ± SEM of three independent experiments. *P < 0.05 indicates significant differences. (b) Representative fluorescence micrographs showed autophagy vacuoles in hepatocytes in the presence or absence of Rap from a pool of at least 10 images. Original magnification ×200, scale bar 50 μm. (c) Quantification of autophagosomes and autolysosomes. *P < 0.05. (d) Quantification of LDH levels in the cultured medium. The data are shown as the means ± SEM of three independent experiments. *P < 0.05 vs. the corresponding vehicle (Veh) group, $^{\#}P$ < 0.05 vs. the corresponding control group.

Original images of cropped blots

Fig. 1b

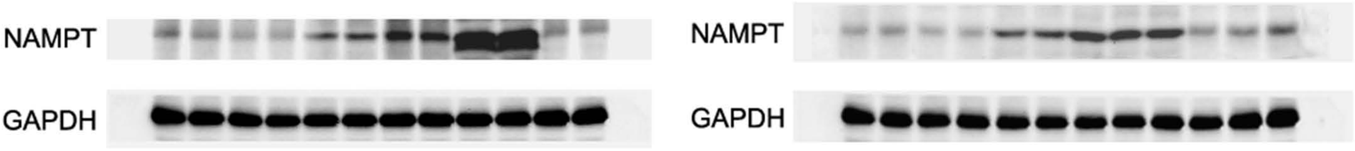

Fig. 1f

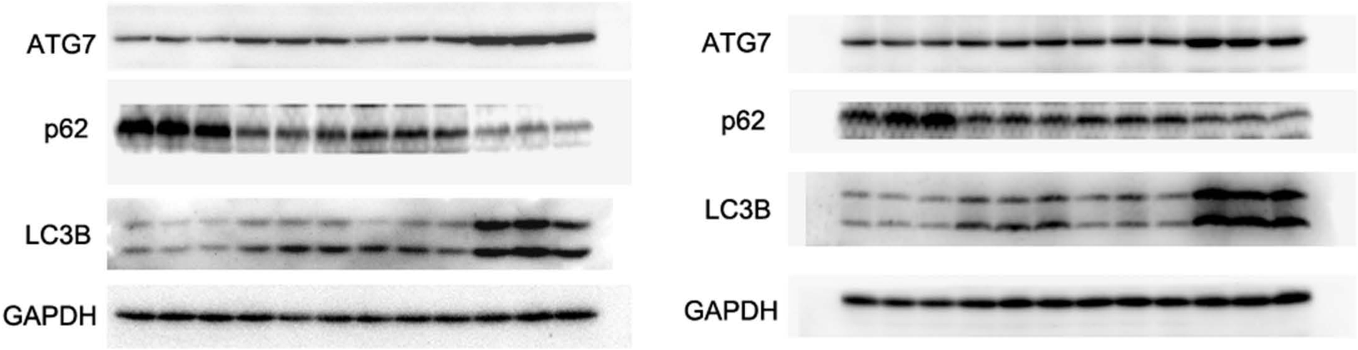

Fig. 1g

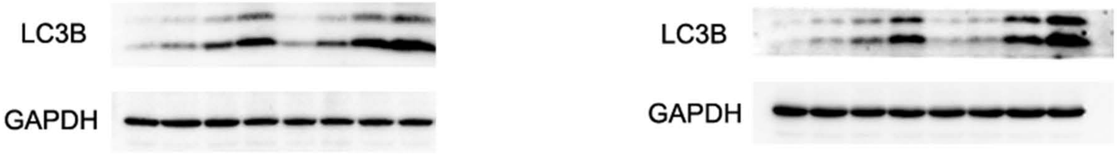

Fig. 2a

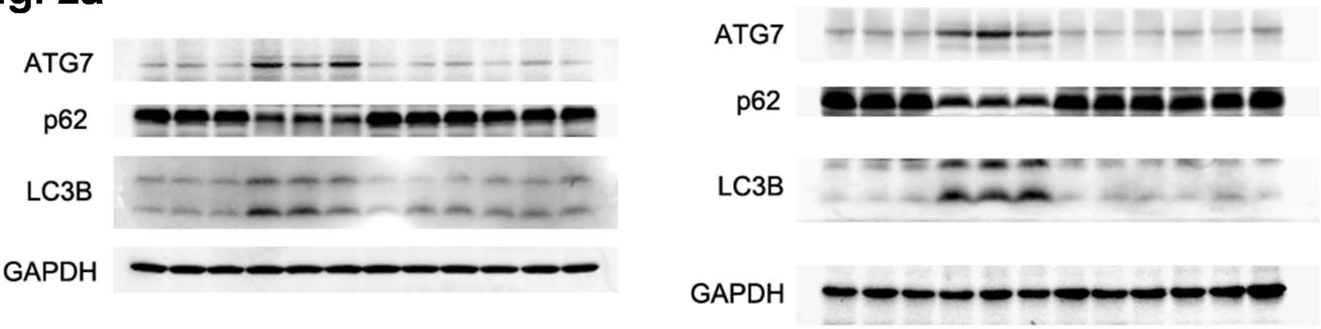

Fig. 2b

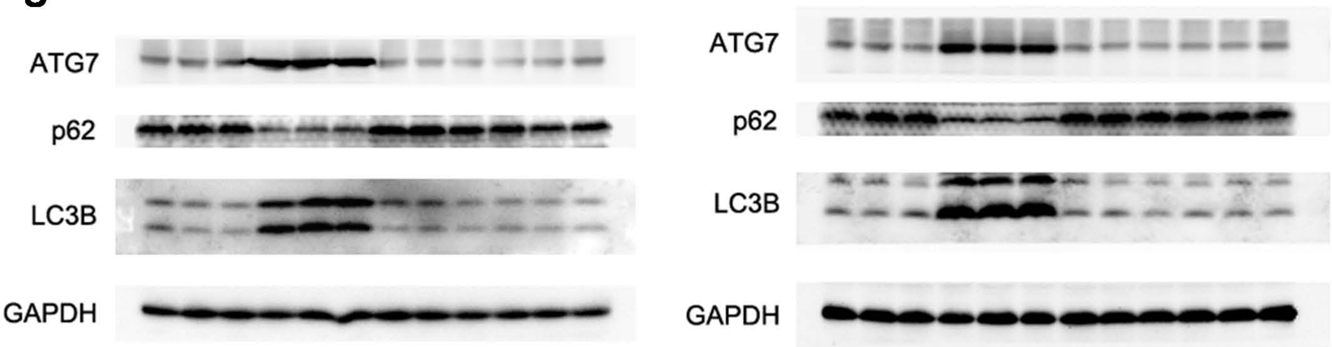

Fig. 3a

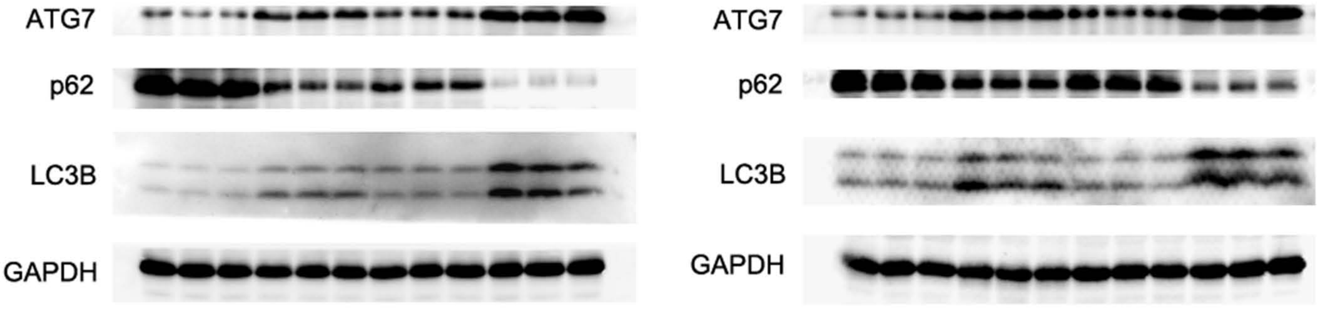

Fig. 4a

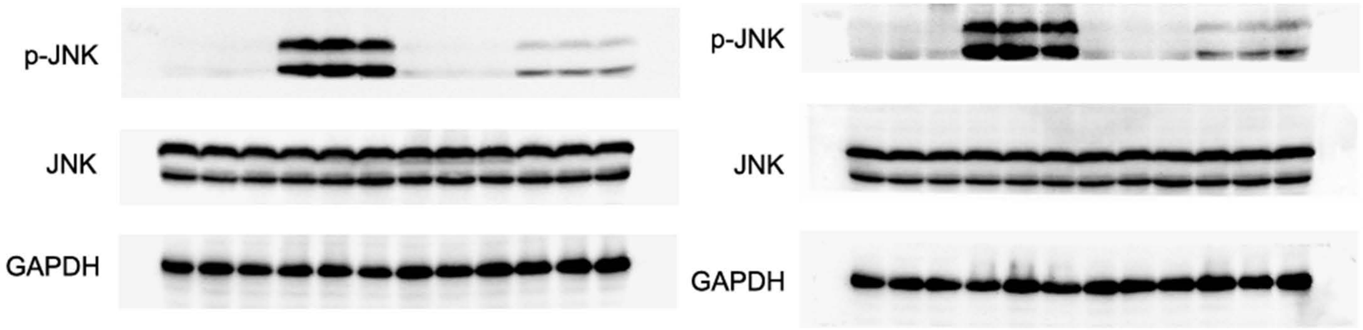

Fig. 4b

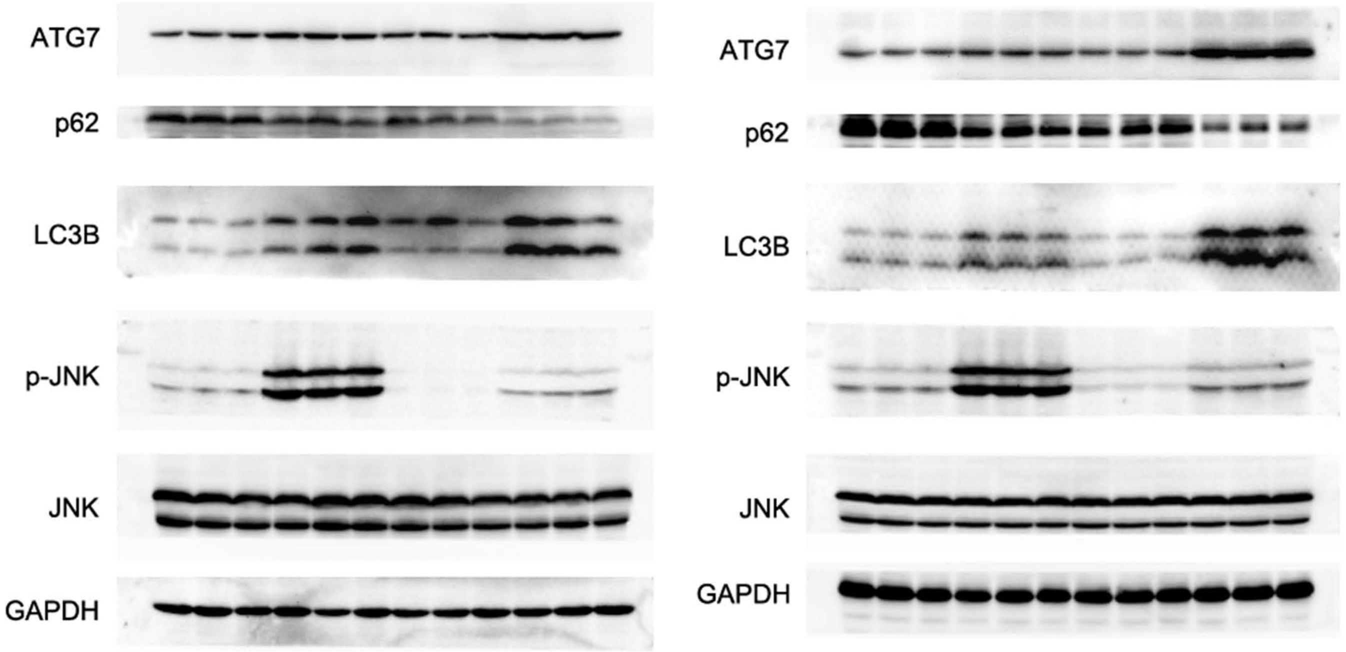

Fig. 5a

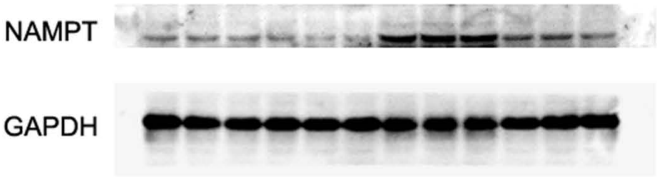

Fig. 5b

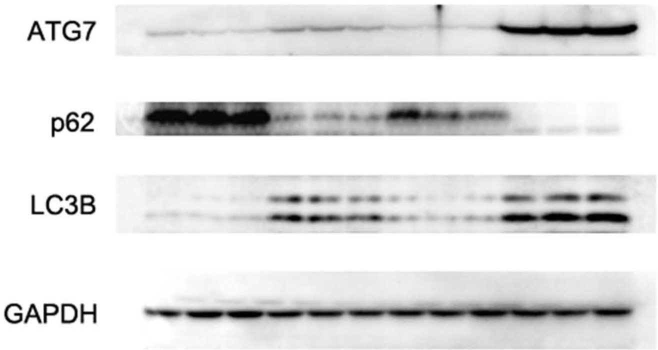

Fig. 5f

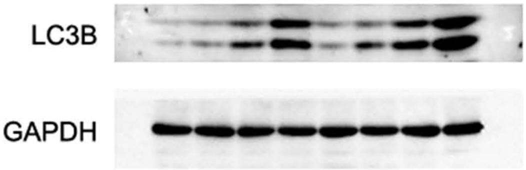

Fig. 6a

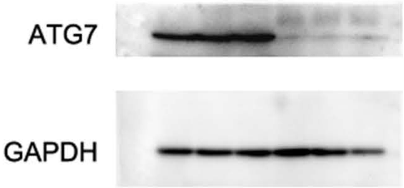

Fig. 7a

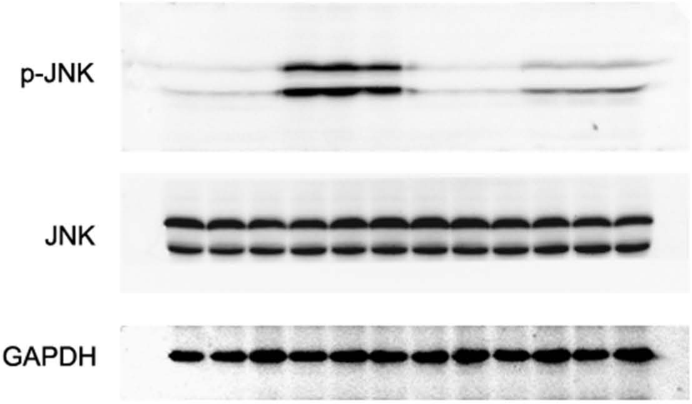

Fig. 7b

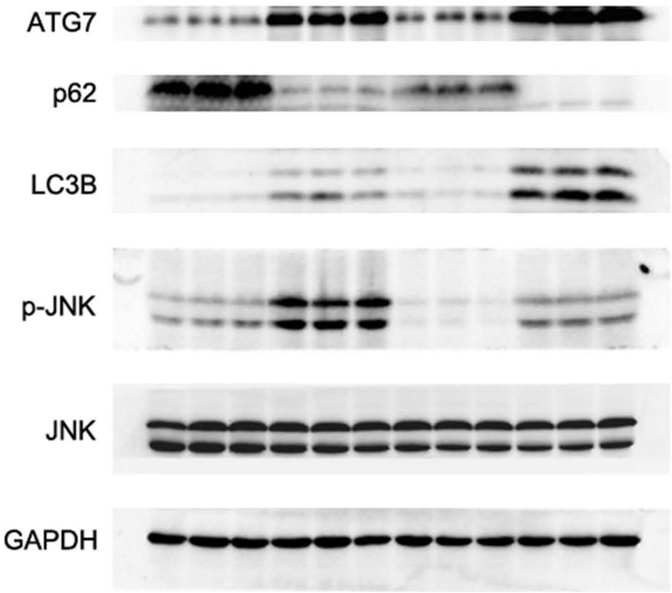

Fig. 7f

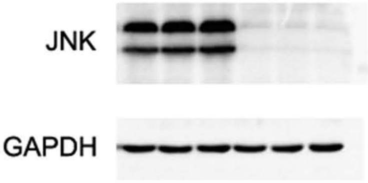

Fig. 7g

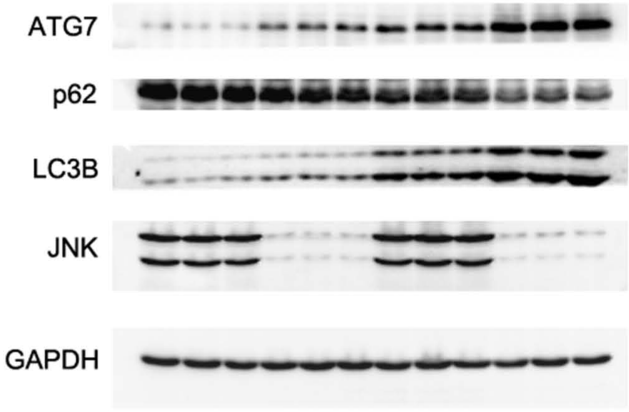

Supplement: Supplementary file 1 — Supplementary information [file 41598_2017_2318_MOESM1_ESM.pdf]
